# Associated factors in the development of rapidly progressive interstitial lung disease in patients with idiopathic inflammatory myopathies: a systematic review and meta-analysis

**DOI:** 10.3389/fimmu.2025.1628928

**Published:** 2025-08-05

**Authors:** Weiwei Yuan, Xuefei Zhou, Yahui Yang, Shijie Zhang, Xing He, Jiaqi Ji

**Affiliations:** ^1^ School of Medicine, University of Electronic Science and Technology of China, Chengdu, China; ^2^ Department of Pediatrics, The First People’s Hospital of Shuangliu District/West China (Airport) Hospital Sichuan University, Chengdu, China; ^3^ Department of Pulmonary and Critical Care Medicine, West China Hospital, Sichuan University, Chengdu, China; ^4^ State Key Laboratory of Respiratory Health and Multimorbidity, West China Hospital, Sichuan University, Chengdu, China; ^5^ Department of Pulmonary and Critical Care Medicine, Sichuan Provincial People’s Hospital, School of Medicine, University of Electronic Science and Technology of China, Chengdu, China

**Keywords:** idiopathic inflammatory myopathy, rapidly progressive interstitial lung disease, development, associated factors, anti-MDA5

## Abstract

**Objectives:**

Interstitial lung disease (ILD), the main pulmonary manifestation of idiopathic inflammatory myopathy (IIM), frequently develops into rapidly progressive ILD (RP-ILD) with significantly worse prognosis. This meta-analysis identified risk and protective factors associated with developing RP-ILD in IIM patients.

**Methods:**

PubMed, Embase, Web of Science, and Scopus (up to October 2024) were searched, analyzing 21 retrospective studies (2,099 patients). Pooled odds ratios (ORs) with 95% confidence intervals (CIs) were collected. Subgroup analysis was performed based on the RP-ILD definition. Sensitivity analysis and publication bias assessments (Egger’s test and trim-and-fill method) were performed.

**Results:**

The associated risk factors for RP-ILD development in IIM patients included age (OR = 1.014, 95% CI: 1.002–1.025), clinically amyopathic dermatomyositis (OR = 3.023, 95% CI: 1.491–6.130), mechanic’s hands (OR = 1.421, 95% CI: 1.054–1.915), fever (OR = 3.090, 95% CI: 1.933–4.939), pulmonary infection (OR = 2.610, 95% CI: 1.457–4.677), anti-melanoma differentiation-associated gene 5 (anti-MDA5) antibodies (OR = 6.044, 95% CI: 4.331–8.435), anti-Ro-52 antibodies (OR = 2.425, 95% CI: 1.807–3.255), and elevated levels of ferritin (OR = 5.844, 95% CI: 4.121–8.287), lactate dehydrogenase (OR = 3.627, 95% CI: 2.406–5.466), erythrocyte sedimentation rate (OR = 1.598, 95% CI: 1.089–2.344), aspartate aminotransferase (OR = 2.666, 95% CI: 1.864–3.814), alanine transaminase (OR = 2.702, 95% CI: 1.737–4.201), and C-reactive protein (OR = 3.366, 95% CI: 2.149–5.274), whereas longer disease duration (OR = 0.790, 95% CI: 0.638–0.977) and dysphagia (OR = 0.773, 95% CI: 0.653–0.916) were potential protective factors.

**Conclusion:**

This meta-analysis of 21 retrospective studies identified potential risk and protective factors associated with RP-ILD development in IIM patients, providing a basis for early identification and management.

**Systematic Review Registration:**

https://inplasy.com/inplasy-2025-4-0059/, identifier INPLASY202540059.

## Introduction

Idiopathic inflammatory myopathy (IIM), collectively known as myositis, is a rare inflammatory systemic disease of unknown cause, characterized by skeletal muscle weakness and chronic inflammation (1). Based on clinical and histopathologic differences, it can be classified into polymyositis (PM), dermatomyositis (DM), and other subtypes such as antisynthetase syndrome (ASyS). IIM often involves multiple organs, including the skin, heart, gastrointestinal tract, and lungs. The main causes of morbidity and mortality in patients with IIM are interstitial lung disease (ILD) (2, 3). The main features of patients with IIM-ILD are inflammatory infiltration and interstitial fibrosis (1). It has been shown that the presence of a clinical ILD is a significant associated factor for poor DM/PM outcomes, even when treated with immunosuppressive agents (4). Rapidly progressive ILD (RP-ILD), in particular, is challenging to treat and has a high mortality rate within a few months, with some patients progressing to RP-ILD with a mortality rate of 33%–66% despite intensive treatment (5). RP-ILD has been recognized as a significant cause of death in IIM patients. Therefore, the systematic identification of RP-ILD development can provide valuable assistance in assessing clinical medication and mortality risk, which is crucial for informed clinical practice.

The development of RP-ILD in patients with IIM has been associated with various factors, including advanced age, elevated serum ferritin levels, and anti-melanoma differentiation-associated gene 5 (anti-MDA5) antibodies. The results of some retrospective studies (6, 7) have found that the development of RP-ILD in patients with IIM is associated with high levels of serum ferritin, older age, anti-MDA5 antibodies, mechanic’s hands, and elevated white blood cell counts. However, there is incomplete consistency of conclusions reported in different studies. Some of the results remain controversial due to the lack of pooled evidence.

Therefore, we assessed the associated factors for RP-ILD development in IIM patients through a pooled analysis to provide further assistance for early clinical recognition and management.

## Materials and methods

The study was conducted in accordance with the Preferred Reporting Items for Systematic Reviews and Meta-analyses (PRISMA) guidelines (8) and registered with INPLASY (http://INPLASY.com) under registration number INPLASY202540059.

### Search strategy

A comprehensive search was carried out for English studies published from the inception date until October 16, 2024, in the Web of Science, PubMed, Embase, and Scopus databases. The search terms were as follows: “rapidly progressive interstitial lung disease”, “Myositis”, “Dermatomyositis”, “Polymyositis”, “Myositis, Inclusion Body”, “Amyopathic dermatomyositis”, “anti-synthetase syndrome”, “RP-ILD”, “DM”, and “PM” (**Supplementary Table 1**).

### Eligibility criteria

The main inclusion criteria included the following: i) retrospective and prospective studies and ii) DM, PM, or clinically amyopathic dermatomyositis (CADM) diagnosis based on the European League Against Rheumatism/American College of Rheumatology (EULAR/ACR) IIM classification criteria (9) or Bohan and Peter’s diagnostic criteria (10). The diagnosis of ASyS was established as positive for one of the five tested anti-synthetase antibodies (Jo-1, PL-7, PL-12, EJ, and OJ), as well as at least one of the triad, including myositis, arthritis, and ILD (11). iii) RP-ILD requires radiological progression on High-resolution computed tomography (HRCT) (new or worsening ground-glass opacities, consolidation, or interstitial abnormalities) within 3 months of respiratory symptom onset, accompanied by progressive worsening of dyspnea, with or without functional decline (PaO_2_ decrease >10 mmHg, Forced vital capacity (FVC) decline >10%) (12–15). iv) Logistic regression modeling was used to obtain the odds ratio (OR) and 95% confidence intervals (CIs) of the associated factors for RP-ILD development in IIM patients; v) studies should be written in English.

The exclusion criteria included the following: i) duplicated literature; ii) case report, conference abstract, review or meta-analysis, animal or cell study, comment or letter, and other types of literature; iii) studies not related to IIM-ILD; iv) studies not reporting RP-ILD as an outcome event; v) data could not be extracted; and vi) literature not in English.

### Variable selection and subgroup analysis

According to the PRISMA declaration process, XZ and YY comprehensively searched the literature in various databases, read the abstract and full text of the literature related to RP-ILD in IIM patients, and selected the associated factors obtained through the analysis of the univariate logistic regression model. The associated factors included in the summary analysis should meet the requirement that at least three pieces of literature provide relevant data. Thresholds of continuous variables in this analysis were directly derived from included retrospective studies, with the minimum reported cut-off values selected for pooled analysis to optimize clinical sensitivity.

We stratified included studies into two subgroups [subgroup 1 (<1-month progression, n = 9) and subgroup 2 (<3-month progression, n = 12)] to evaluate the methodological heterogeneity in RP-ILD progression definitions (**Supplementary Table 2**).

### Quality assessment (risk of bias) and data extraction

XZ and YY independently screened all studies, with conflicts resolved by an arbitrator (JJ). The Newcastle–Ottawa Scale (NOS) was applied to assess the quality of the included literature using a semi-quantitative scoring system, with a maximum score of nine stars (12); ratings of 1–3 stars, 4–6 stars, and 7–9 stars were defined as low, medium, and high quality, respectively. The literature quality assessment was independently completed by SZ and XH (**Supplementary Table 3**). The relevant data from the included literature were extracted independently by XZ and YY, including study number, author names and publication dates, type of the studies, total number of patients with IIM-ILD and total number of patients with RP-ILD in IIM patients, country of origin, time span of the disease in the included patients, subtypes of IIM, and factors associated with the development of RP-ILD in IIM patients (**Table 1**).

**Table 1:** Baseline characteristics of the 21 included studies.

No.	Author	Publication year	Study type	nTotal/nRP-ILD	Country	Time span	IIM subtypes	Subgroup^#^	Associated factors (threshold value)
1	Zhang Y et al. (23)	2022	Retrospective study	55/17	China	2013–2021	DM/PM/CADM	2	Ferritin (>1,000 μg/L), anti-MDA5
2	Li Y et al. (6)	2021	Retrospective study	326/86	China	2010–2019	DM/CADM	2	Female, DM, Gottron papules, mechanic’s hands, fever, heliotrope rash, dysphagia, V sign, shawl sign, skin ulceration, periungual erythema, muscle weakness, anti-MDA5, anti-Ro-52, anti-Jo-1, anti-TIF1-γ, anti-ARS, anti-Ku, CK (elevated), CRP (elevated > 2.78 mg/L), LDH (elevated), ESR (elevated), AST (elevated), ALT (elevated), age
3	Li Y et al. (29)	2024	Retrospective study	103/44	China	NA	DM (anti-MDA5^+^)	1	Dyspnea, disease duration
4	Tsai HC et al. (19)	2024	Retrospective study	39/22	China	2018–2022	DM/PM (anti-MDA5^+^)	2	Ferritin (>336 ng/mL), female, anti-Ro-52, LDH (>280 U/L), AST (>36 U/L)
5	Zhang Y et al. (7)	2020	Retrospective study	44/10	China	2004–2018	ASyS	2	Female, Gottron papules, dyspnea, mechanic’s hands, fever, heliotrope rash, dysphagia, V sign, shawl sign*, muscle weakness, arthritis, pulmonary infection, anti-Ro-52, disease duration, age
6	Liang J et al. (16)	2021	Retrospective study	151/29	China	2018–2020	DM/PM/CADM	1	Female, DM, CADM, dysphagia, pulmonary infection, anti-MDA5, anti-Ro-52, anti-Jo-1, Anti-TIF1-γ, anti-Ku, age
7	Zhu D et al. (25)	2021	Retrospective study	41/16	China	2010–2019	CADM	2	Age
8	Shi Y et al. (18)	2024	Retrospective study	39/20	China	NA	DM (anti-MDA5^+^)/ASyS	1	Ferritin (>336 ng/mL), female, Gottron papules, mechanic’s hands, heliotrope rash, V sign, shawl sign, skin ulceration, periungual erythema, muscle weakness, arthritis, CK (>200 U/L), CRP (>8 mg/L), ESR (>21 mm/h), age
9	Shen Y et al. (17)	2020	Retrospective study	102/29	China	2005–2016	DM	2	Ferritin (≥417.7 ng/mL), female, Gottron papules, mechanic’s hands, heliotrope rash, dysphagia, skin ulceration, muscle weakness, arthritis, anti-MDA5, anti-ARS, age
10	Yang Q et al. (31)	2021	Retrospective study	81/35	China	2018–2019	DM/CADM/PM (anti-MDA5^+^)	1	Ferritin (≥1,500 ng/mL), CRP (≥13 mg/L)
11	Karino K et al. (26)	2020	Retrospective study	30/10	Japan	2014–2019	DM	2	Anti-MDA5, age
12	Xu Y et al. (21)	2016	Retrospective study	40/11	China	NA	CADM	2	Ferritin (>2,000 ng/mL), skin ulceration, anti-MDA5, CRP (>50 μg/L)
13	Li M et al. (27)	2023	Retrospective study	73/21	China	2017–2021	DM (anti-MDA5^+^)	2	Female, anti-Ro-52, LDH (>356.15 U/L), AST (>71.27 U/L), ALT (>27.75 U/L), disease duration^*^, age
14	He W et al. (13)	2024	Retrospective study	119/44	China	2014–2022	DM (anti-MDA5^+^)	2	Female, Gottron papules, dyspnea, mechanic’s hands, fever, heliotrope rash, V sign, skin ulceration, periungual erythema, arthritis, age
15	Wang K et al. (20)	2022	Retrospective study	257/41	China	2017–2020	DM	1	Female, Gottron papules, mechanic’s hands, Heliotrope rash, shawl sign, skin ulceration, periungual erythema, muscle weakness, arthritis, anti-MDA5, anti-Ro-52, anti-ARS, disease duration, age
16	Zhang H et al. (22)	2023	Retrospective study	71/17	China	2018–2022	DM	2	Female, fever, periungual erythema, anti-MDA5, age
17	Zhu Y et al. (24)	2022	Retrospective study	41/21	China	2019–2021	DM (anti-MDA5^+^)	1	Ferritin (>336.2 ng/mL), female, Gottron papules, mechanic’s hands, heliotrope rash, V sign, shawl sign, skin ulceration, periungual erythema, muscle weakness, arthritis, ESR (>21 mm/h)
18	Li C et al. (14)	2024	Retrospective study	50/31	China	2017–2022	DM	1	Ferritin (≥389.5 ng/mL), female, Gottron papules, arthritis, anti-MDA5, anti-ARS, age
19	Liang J et al. (15)	2021	Retrospective study	61/21	China	2017–2022	DM/PM/CADM	1	Female, DM, CADM, pulmonary infection, anti-MDA5, anti-Ro-52, anti-Jo-1, anti-TIF1-γ, anti-Ku, disease duration, age
20	Sagawa T et al. (30)	2019	Retrospective study	29/15	Japan	2008–2017	DM	1	CADM
21	Guo L et al. (28)	2023	Retrospective study	347/101	China	2018–2021	DM/ASyS	2	Ferritin (≥823 ng/mL), Gottron papules, mechanic’s hands, heliotrope rash, anti-Ro-52, LDH (≥282.5 U/L), CK (maximum ≥47.5 U/L), CRP (≥2.86 mg/dL), ESR (> 29.5 mm/h), AST (≥52.5 IU/L^@^), ALT (≥39.5 IU/L), age

^#^According to the definition of RP-ILD: subgroup 1: RP-ILD progression time <1 month; subgroup 2: RP-ILD progression time <3 months.

*These data were excluded based on sensitivity analysis due to their significant impact on the robustness of the aggregated analysis results.

@: U/L (unit per liter) is a common unit of enzyme activity; IU/L refers to the international unit per liter, which is also the international unit of enzyme activity. In general, the two are often considered equivalent.

NA, not available; IIM, idiopathic inflammatory myopathy; RP-ILD, rapidly progressive interstitial lung disease; DM, dermatomyositis; PM, polymyositis; CADM: clinically amyopathic dermatomyositis; ASyS, antisynthetase syndrome; MDA5, melanoma differentiation-associated 5; TIF-1γ, translation initiation factor-1γ; CK, creatine kinase; CRP, C-reactive protein; LDH, lactate dehydrogenase; ESR: erythrocyte sedimentation rate; AST, aspartate aminotransferase; ALT, alanine transaminase.

### Data synthesis

The OR and 95% CI of the associated factors were collected as statistical effect sizes, and Cochran’s Q statistic and inconsistency value (I^2^) were used to test the heterogeneity of the included studies. If p < 0.05 and I^2^ ≥ 50%, heterogeneity was significant, and pooled analyses were performed using the random-effects model and DerSimonian–Laird (DL) method. Otherwise, the fixed-effects model and inverse variance (IV) method were used. Pooled subgroup analyses were performed for different associated factors. Excluding one category of study at a time method was utilized for sensitivity analysis. If there was no significant effect on the results after excluding a study, it means that our results were stable and reliable. Publication bias was assessed using a combination of Egger’s test and the rim-and-fill method. The Stata 16.0 software, the meta package, and RStudio 4.4.3 were used for meta-analysis, with statistical significance set at p < 0.05.

## Result

**Figure 1:**
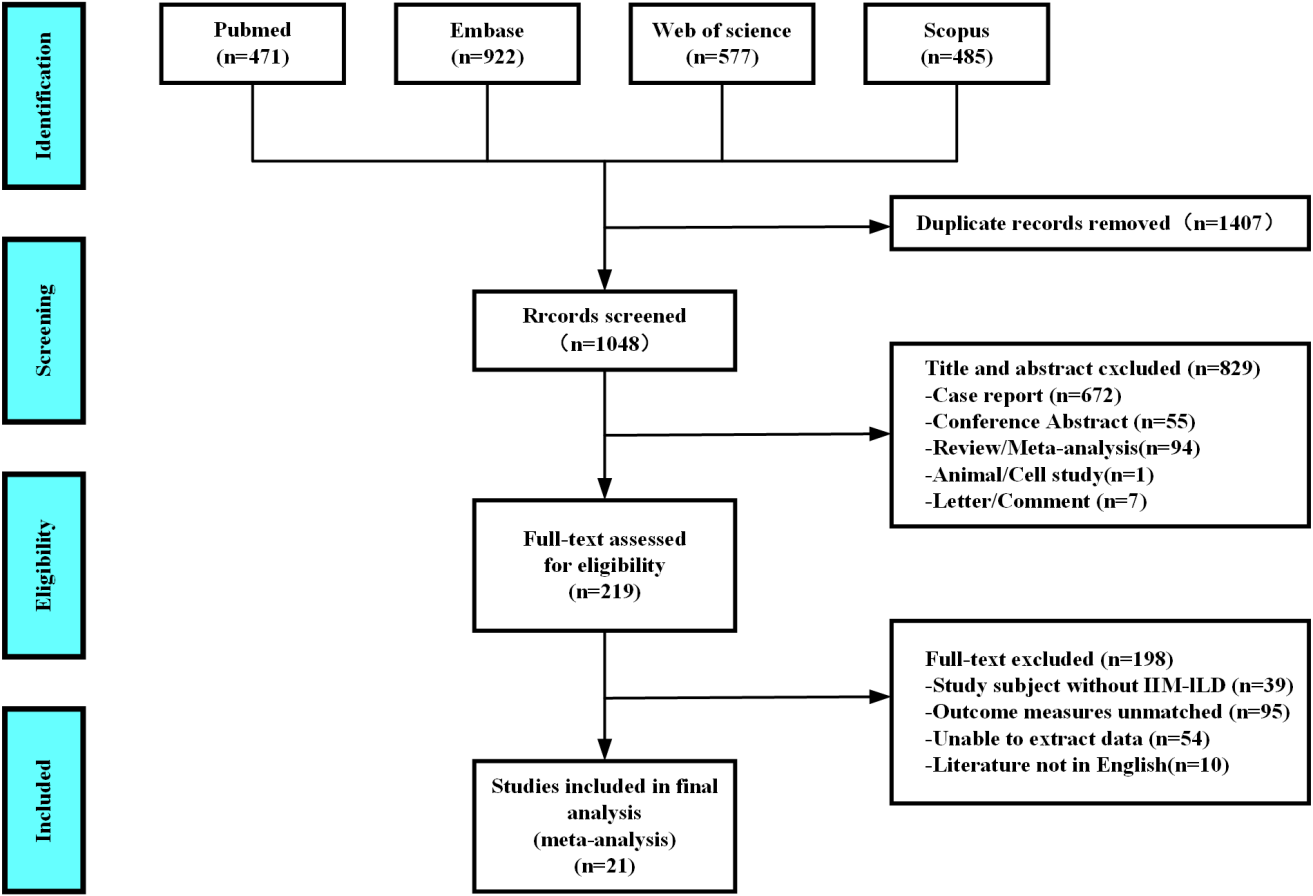
Diagram of the preferred reporting items for systematic reviews and meta-analyses (PRISMA).

As shown in **Figure 1**, after a thorough evaluation of the abstracts and full texts of 2,455 studies on IIM patients with RP-ILD, we excluded 1,407 duplicate studies, 672 case reports, 55 conference abstracts, 94 reviews or meta-analyses, one animal or cell study, seven letters or comments, 39 studies not related to IIM-ILD, 95 that did not use RP-ILD as an outcome, 54 with data that could not be extracted, and 10 not in English, resulting in the inclusion of 2,099 IIM patients from 21 studies for the pooled analysis of associated factors for RP-ILD development.

All included studies were retrospective in design. All studies were conducted in Asia, including China (n = 19) and Japan (n = 2). Associated factors for RP-ILD development in IIM patients were pooled and analyzed, including demographic characteristics [female (6, 13–24), age (6, 7, 13–18, 20, 22, 25–28), and disease duration (7, 15, 20, 27, 29)], IIM subtype [DM (6, 15, 16) and CADM (15, 16, 30)], skin manifestations [Gottron papules (6, 7, 13, 14, 17, 18, 20, 24, 28), heliotrope rash (6, 7, 13, 17, 18, 20, 24, 28), shawl sign (6, 7, 18, 20, 24), skin ulceration (6, 13, 17, 18, 20, 21, 24), and periungual erythema (6, 13, 18, 20, 22, 24)], other clinical manifestations [dyspnea (7, 13, 29), mechanic’s hands (6, 7, 13, 17, 18, 20, 24, 28), fever (6, 7, 13, 22), dysphagia (6, 7, 16, 17), V sign (6, 7, 13, 18, 24), muscle weakness (6, 7, 17, 18, 20, 24), arthritis (6, 13, 14, 17, 18, 20, 24), and pulmonary infection (7, 15, 16)] and antibody positivity [anti-MDA5 antibody (6, 14–17, 20–23, 26), anti-Ro-52 antibody (6, 7, 15, 16, 19, 20, 28), anti-Jo-1 antibody (6, 15, 16), anti-TIF1-γ antibody (6, 15, 16), anti-ARS antibody (6, 14, 17, 20), and anti-Ku antibody (6, 15, 16)], and laboratory data [ferritin (>336 ng/mL) (14, 17–19, 21, 23, 24, 28, 31), creatine kinase (CK) (≥47.5 U/L) (6, 18, 28), C-reactive protein (CRP) (>0.05 mg/L) (6, 18, 21, 28, 31), lactate dehydrogenase (LDH) (>280 U/L) (6, 19, 27, 28), erythrocyte sedimentation rate (ESR) (>21 mm/h) (6, 18, 24, 28), aspartate aminotransferase (AST) (>36 U/L) (6, 19, 21, 28), and alanine transaminase (ALT) (>27.5 U/L) (6, 27, 28)]. The characteristics of all studies are presented in **Table 1**. After evaluating the quality of the literature using the NOS, all 21 studies were classified as high-quality (**Supplementary Table 3**).

### Pooled analysis of factors associated with RP-ILD development in IIM patients: female sex, disease duration, and age

The heterogeneity results indicated that female sex (I^2^ = 0%, p = 0.995) and age (I^2^ = 48.6%, p = 0.021) showed no significant heterogeneity. Using the fixed-effects model and IV method for analysis, the pooled results revealed that age (OR = 1.014, 95% CI: 1.002–1.025, p = 0.020) was an associated risk factor for RP-ILD development in IIM patients, while female sex (OR = 1.225, 95% CI: 0.937–1.601, p = 0.138) showed no significant association. The results of the subgroup analysis of age showed that there was a significant association between age and the risk of RP-ILD development [subgroup 1 (<1 month): OR = 1.010, p = 0.217; subgroup 2 (<3 months): OR = 1.024, p = 0.124].

**Figure 2:**
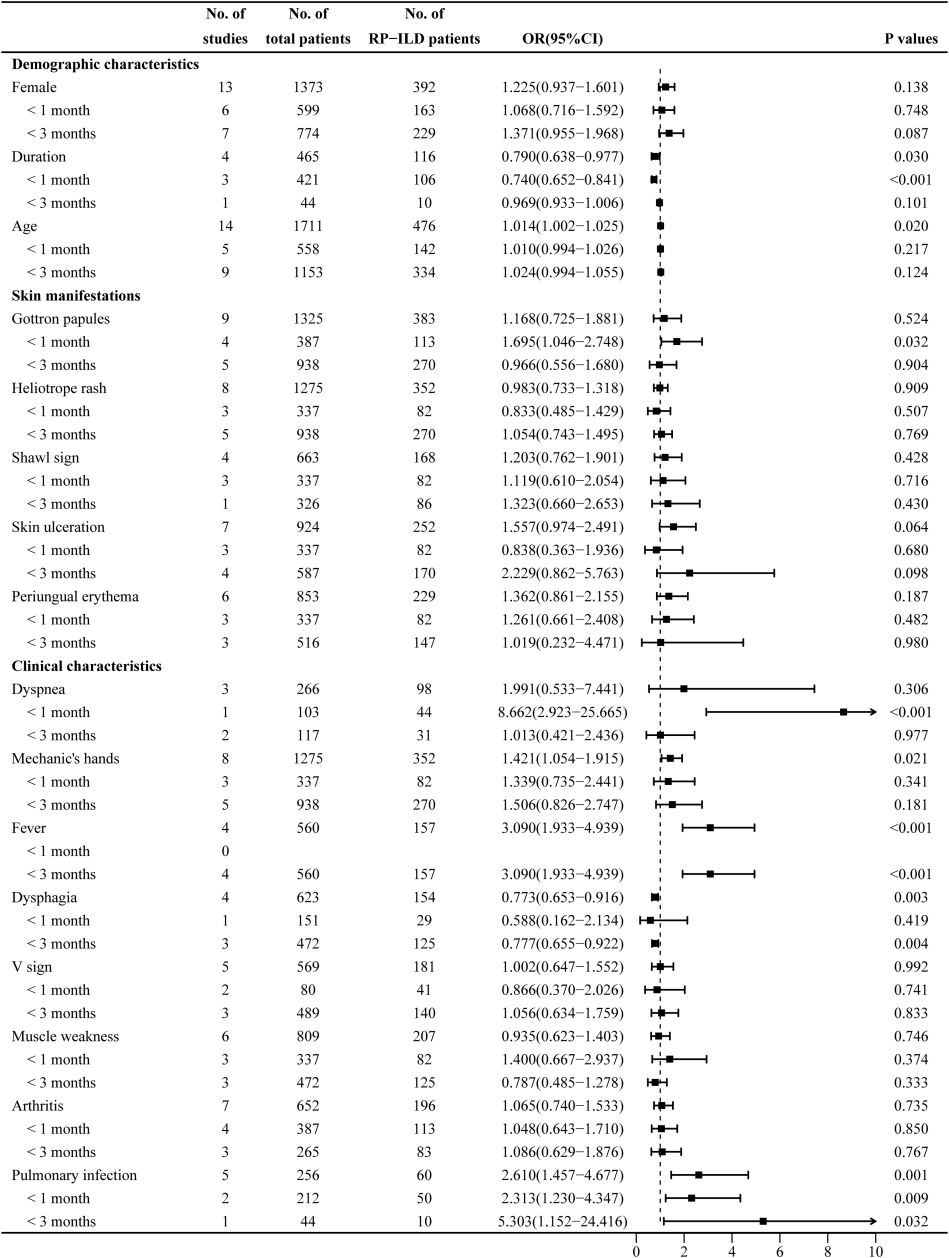
Combined forest plot 1: associated factors for the development of RP-ILD in IIM patients (demographic characteristics, skin manifestations, and clinical characteristics). RP-ILD, rapidly progressive interstitial lung disease; IIM, idiopathic inflammatory myopathy.

The heterogeneity results indicated that disease duration (I^2^ = 84.6%, p < 0.001) showed significant heterogeneity. Using the random-effects model and the DL method for analysis, the pooled results revealed that longer disease duration (OR = 0.790, 95% CI: 0.638–0.977, p = 0.030) was a potential protective factor against RP-ILD development in IIM patients. The results of the subgroup analysis showed that longer disease duration had a potential protective association in subgroup 1 (OR = 0.740, p < 0.001), but had no significant association in subgroup 2 (OR = 0.969, p = 0.101) (**Figure 2**).

### Pooled analysis of factors associated with RP-ILD development in IIM patients: Gottron papules, heliotrope rash, shawl sign, skin ulceration, and periungual erythema

The heterogeneity results indicated that heliotrope rash (I^2^ = 0%, p = 0.727), shawl sign (I^2^ = 0%, p = 0.485), skin ulceration (I^2^ = 48.8%, p = 0.069), and periungual erythema (I^2^ = 42.9%, p = 0.119) showed no significant heterogeneity. Using the fixed-effects model and IV method for analysis, the pooled results revealed that heliotrope rash (OR = 0.983, 95% CI: 0.733–1.318, p = 0.909), shawl sign (OR = 1.203, 95% CI: 0.762–1.901, p = 0.428), skin ulceration (OR = 1.557, 95% CI: 0.974–2.491, p = 0.064), and periungual erythema (OR = 1.362, 95% CI: 0.861–2.155, p = 0.187) showed no significant association.

The heterogeneity results indicated that Gottron papules (I^2^ = 72.3%, p < 0.001) showed significant heterogeneity. Using a random-effects model and the DL method for analysis, the pooled results revealed that Gottron papules (OR = 1.168, 95% CI: 0.725–1.881, p = 0.524) had no significant association with RP-ILD in IIM patients. The results of the subgroup analysis showed that Gottron papules were an associated risk factor for RP-ILD development in IIM patients in subgroup 1 (OR = 1.695, 95% CI: 1.046–2.748, p = 0.032), while there was no significant association in subgroup 2 (OR = 0.966, p = 0.904) (**Figure 2**).

### Pooled analysis of factors associated with RP-ILD development in IIM patients: dyspnea, Mechanic’s hands, fever, dysphagia, V sign, muscle weakness, arthritis, and pulmonary infection

The heterogeneity results indicated that mechanic’s hands (I^2^ = 28.3%, p = 0.203), fever (I^2^ = 50.2%, p = 0.110), dysphagia (I^2^ = 0%, p = 0.915), V sign (I^2^ = 0%, p = 0.973), muscle weakness (I^2^ = 46.5%, p = 0.096), arthritis (I^2^ = 0%, p = 0.940), and pulmonary infection (I^2^ = 0%, p = 0.616) showed no significant heterogeneity. Using the fixed-effects model and IV method for analysis, the pooled result revealed that mechanic’s hands (OR = 1.421, 95% CI: 1.054–1.915, p = 0.021), fever (OR = 3.090, 95% CI: 1.933–4.939, p < 0.001), and pulmonary infection (OR = 2.610, 95% CI: 1.457–4.677, p = 0.001) were associated risk factors for RP-ILD development in IIM patients; dysphagia (OR = 0.773, 95% CI: 0.653–0.916, p = 0.003) was a potential protective factor; and V sign (OR = 1.002, 95% CI: 0.647–1.552, p = 0.992), muscle weakness (OR = 0.935, 95% CI: 0.623–1.403, p = 0.746), and arthritis (OR = 1.065, 95% CI: 0.740–1.533, p = 0.735) showed no significant association. The results of the subgroup analysis showed that there was no significant association between mechanic’s hands and the risk of RP-ILD development (subgroup 1: OR = 1.339, p = 0.341; subgroup 2: OR = 1.506, p = 0.181). In subgroup 2, dysphagia was a potential protective factor (OR = 0.777, p = 0.004), but there was no significant association in subgroup 1 (OR = 0.588, p = 0.419).

The heterogeneity results indicated that dyspnea (I^2^ = 91.7%, p < 0.001) showed significant heterogeneity. Using a random-effects model and the DL method for analysis, the pooled results revealed that there was no significant association between dyspnea (OR = 1.991, 95% CI: 0.533–7.441, p = 0.306) and RP-ILD development in IIM patients. The results of the subgroup analysis showed that dyspnea was an associated risk factor for RP-ILD development in IIM patients in subgroup 1 (OR = 8.662, p < 0.001), while there was no significant association in subgroup 2 (OR = 1.013, p = 0.977) (**Figure 2**).

### Pooled analysis of factors associated with RP-ILD development in IIM patients: DM and CADM subtypes

**Figure 3:**
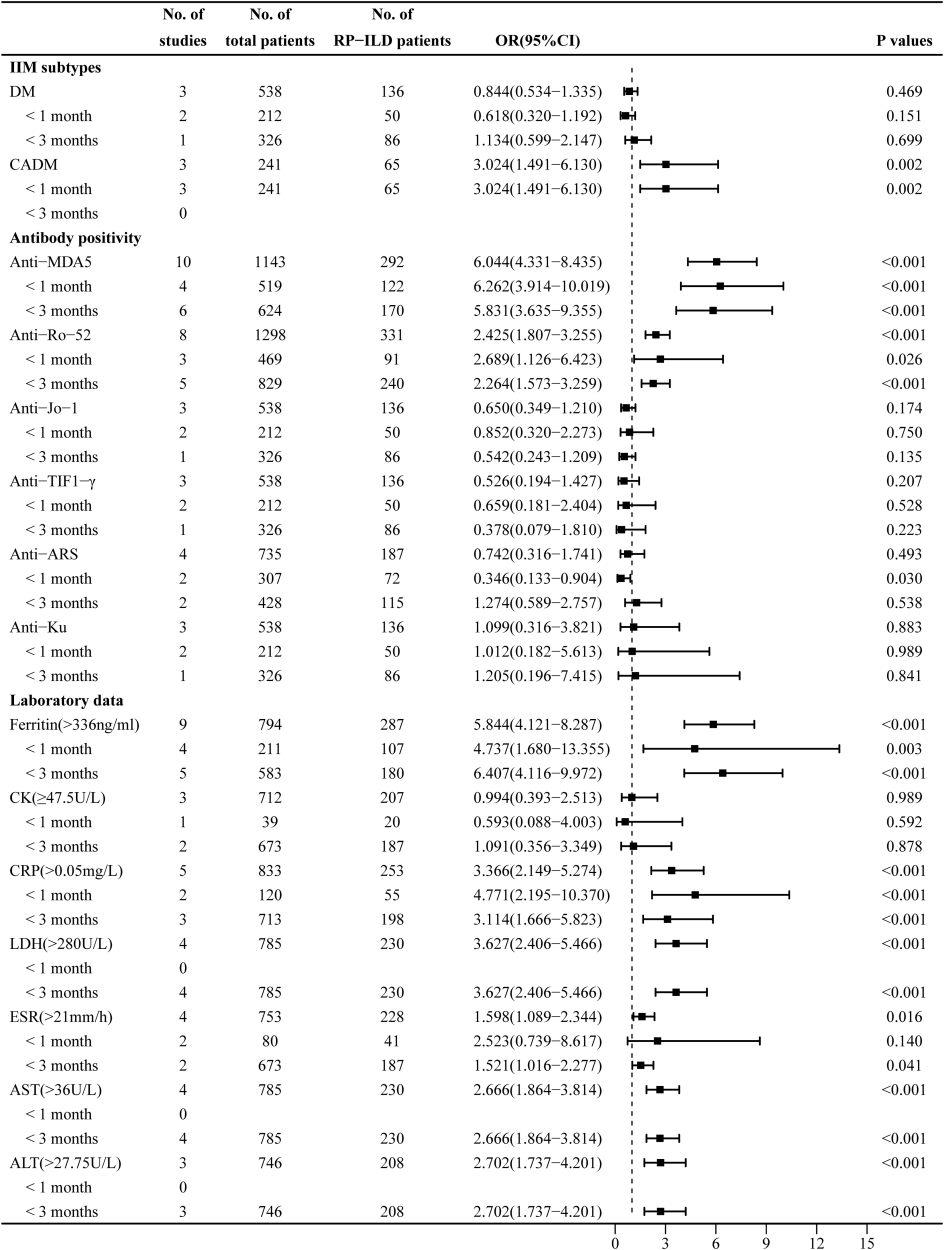
Combined forest plot 2: associated factors for the development of RP-ILD in IIM patients (IIM subtypes, antibody positivity, and laboratory data). RP-ILD, rapidly progressive interstitial lung disease; IIM, idiopathic inflammatory myopathy.

The heterogeneity results indicated that DM (I^2^ = 0%, p = 0.375) and CADM (I^2^ = 0%, p = 0.806) showed no significant heterogeneity. Using the fixed-effects model and IV method for analysis, the pooled results revealed that CADM (OR = 3.024, 95% CI: 1.491–6.130, p = 0.002) was an associated risk factor for RP-ILD development in IIM patients, while DM (OR = 0.844, 95% CI: 0.534–1.335, p = 0.469) had no significant association. The results of the subgroup analysis showed that CADM was an associated risk factor for RP-ILD development in IIM patients in subgroup 1 (OR = 3.024, p = 0.002), while there was no significant association between DM and the risk of RP-ILD development (subgroup 1: OR = 0.618, p = 0.151; subgroup 2: OR = 1.134, p = 0.699) (**Figure 3**).

### Pooled analysis of factors associated with RP-ILD development in IIM patients: anti-MDA5, anti-Ro-52, anti-Jo-1, anti-TIF1-γ, anti-ARS, and anti-Ku antibodies

The heterogeneity results indicated that there was no significant heterogeneity in antibodies including anti-MDA5 antibody (I^2^ = 22.6%, p = 0.235), anti-Ro-52 antibody (I^2^ = 37.9%, p = 0.127), anti-Jo-1 antibody (I^2^ = 0%, p = 0.685), and anti-TIF1-γ antibody (I^2^ = 0%, p = 0.864). Using the fixed-effects model and IV method for analysis, the pooled results revealed that anti-MDA5 antibody (OR = 6.044, 95% CI: 4.331–8.435, p < 0.001) and anti-Ro-52 antibody (OR = 2.425, 95% CI: 1.807–3.255, p < 0.001) were associated risk factors for RP-ILD development in IIM patients, while anti-Jo-1 antibody (OR = 0.650, 95% CI: 0.349–1.210, p = 0.174), anti-TIF1-γ antibody (OR = 0.526, 95% CI: 0.194–1.427, p = 0.207), and anti-Ku antibody (OR = 1.099, 95% CI: 0.316–3.821, p = 0.883) had no significant association. The results of the subgroup analysis showed that there were significant associations between anti-MDA5 antibody and the risk of RP-ILD development (subgroup 1: OR = 6.262, p < 0.001; subgroup 2: OR = 5.831, p < 0.001) and between anti-Ro-52 antibody and the risk of RP-ILD development (subgroup 1: OR = 2.689, p = 0.026; subgroup 2: OR = 2.264, p < 0.001).

The heterogeneity results indicated that anti-ARS antibody (I^2^ = 69.2%, p = 0.021) showed significant heterogeneity. Using a random-effects model and the DL method for analysis, the pooled results revealed that anti-ARS (OR = 0.742, 95% CI: 0.316–1.741, p = 0.493) had no significant association. The results of the subgroup analysis showed that anti-ARS had a potential protective association in subgroup 1 (OR = 0.346, p = 0.030), but had no significant association in subgroup 2 (OR = 1.274, p = 0.538) (**Figure 3**).

### Pooled analysis of factors associated with RP-ILD development in IIM patients: ferritin, CK, CRP, ESR, LDH, ALT, and AST

The heterogeneity results indicated that ferritin (>336 ng/mL) (I^2^ = 34%, p = 0.146), CRP (>0.05 mg/L) (I^2^ = 24.9%, p = 0.225), LDH (>280 U/L) (I^2^ = 39.3%, p = 0.176), ESR (>21 mm/h) (I^2^ = 16.3%, p = 0.310), AST (>36 U/L) (I^2^ = 0%, p = 0.513), and ALT (>27.75 U/L) (I^2^ = 27.2%, p = 0.253) showed no significant heterogeneity. Using the fixed-effects model and IV method for analysis, the pooled results revealed that ferritin (>336 ng/mL) (OR = 5.844, 95% CI: 4.121–8.287, p < 0.001), CRP (>0.05 mg/L) (OR = 3.366, 95% CI: 2.149–5.274, p < 0.001), LDH (>280 U/L) (OR = 3.627, 95% CI: 2.406–5.466, p < 0.001), ESR (>21 mm/h) (OR = 1.598, 95% CI: 1.089–2.344, p = 0.016), AST (>36 U/L) (OR = 2.666, 95% CI: 1.864–3.814, p < 0.001), and ALT (>27.75 U/L) (OR = 2.702, 95% CI: 1.737–4.201, p < 0.001) were associated risk factors for RP-ILD development in IIM patients. The results of the subgroup analysis showed that ferritin (>336 ng/mL) (subgroup 1: OR = 4.737, p = 0.003; subgroup 2: OR = 6.407, p < 0.001) and CRP (>0.05 mg/L) (subgroup 1: OR = 4.771, p < 0.001; subgroup 2: OR = 3.114, p < 0.001) were associated risk factors for RP-ILD development in IIM patients, and ESR (>21 mm/h) was an associated risk factor particularly in subgroup 2 (OR = 1.521, p = 0.041).

The heterogeneity results indicated that CK (≥47.5 U/L) (I^2^ = 69.7%, p = 0.037) showed significant heterogeneity. Using a random-effects model and the DL method for analysis, the pooled results revealed that CK (≥47.5 U/L) (OR = 0.994, 95% CI: 0.393–2.513, p = 0.989) had no association with RP-ILD development (**Figure 3**).

### Sensitivity analysis and publication bias

The results of the sensitivity analysis showed stable results in all our studies (**Supplementary Figures 1**–**6**). The results of the heterogeneity analysis showed that, among all subgroup factors, there was no significant heterogeneity in the included literature for any subgroup factor, except for Gottron papules, dyspnea, CK (≥47.5 U/L), CRP (>0.05 mg/L), and disease duration. The included studies were found to be largely free of publication bias by Egger’s test (p > 0.05) (**Table 2**). Next, we corrected all pooled studies for associated factors on RP-ILD development in IIM patients using the trim-and-fill method, and our results showed that ferritin (>336 ng/mL) (n = 1), Gottron papules (n = 1), fever (n = 2), anti-MDA5 antibody (n = 1), anti-Ro-52 antibody (n = 2), anti-Jo-1 antibody (n = 2), anti-ARS antibody (n = 2), anti-Ku antibody (n = 1), CRP (>0.05 mg/L) (n = 3), ALT (>27.75 U/L) (n = 1), and age (n = 3) showed symmetrical funnel plots and unchanged statistics after supplementing with the corresponding literature. The funnel plots for the remaining parameters were also symmetrical, indicating that our included studies were largely free of publication bias (**Supplementary Figures 7**–**12**).

**Table 2:** Meta-analysis results of the associated factors in IIM patients with RP-ILD development.

Classification	Factors	No. of studies	No. of total patients	No. of RP-ILD	Effects model	I^2^ (%)	p-Value (heterogeneity)	OR (95% CI)	p-Value (OR)	Egger’s test		Influence on RP-ILD development
										t	p-Value	
Demographic characteristics	Female	13	1,373	392	Fixed	0	0.995	1.225 (0.937–1.601)	0.138	−1.33	0.211	NS
	<1 month	6	599	163	Fixed	0	0.996	1.068 (0.716–1.592)	0.748			NS
	<3 months	7	774	229	Fixed	0	0.929	1.371 (0.955–1.968)	0.087			NS
	Disease duration	4	465	116	Random	84.6	<0.001	0.790 (0.638–0.977)	0.030	−4.00	0.057	PF
	<1 month	3	421	106	Fixed	44.4	0.166	0.740 (0.652–0.841)	<0.001			PF
	<3 months	1	44	10	NA	NA	NA	0.969 (0.933–1.006)	0.101			NS
	Age	14	1,711	476	Fixed	48.6	0.021	1.014 (1.002–1.025)	0.020	2.57	0.024	RF
	<1 month	5	558	142	Fixed	0	0.467	1.010 (0.994–1.026)	0.217			NS
	<3 months	9	1,153	334	Random	62.6	0.006	1.024 (0.994–1.055)	0.124			NS
Skin manifestations	Gottron papules	9	1,325	383	Random	72.3	<0.001	1.168 (0.725–1.881)	0.524	2.66	0.032	NS
	<1 month	4	387	113	Fixed	38.4	0.181	1.695 (1.046–2.748)	0.032			RF
	<3 months	5	938	270	Random	73.2	0.005	0.966 (0.556–1.680)	0.904			NS
	Heliotrope rash	8	1,275	352	Fixed	0	0.727	0.983 (0.733–1.318)	0.909	−0.15	0.888	NS
	<1 month	3	337	82	Fixed	0	0.547	0.833 (0.485–1.429)	0.507			NS
	<3 months	5	938	270	Fixed	0	0.604	1.054 (0.743–1.495)	0.769			NS
	Shawl sign	4	663	168	Fixed	0	0.485	1.203 (0.762–1.901)	0.428	−3.76	0.064	NS
	<1 month	3	337	82	Fixed	13.9	0.313	1.119 (0.610–2.054)	0.716			NS
	<3 months	1	326	86	NA	NA	NA	1.323 (0.660–2.653)	0.430			NS
	Skin ulceration	7	924	252	Fixed	48.8	0.069	1.557 (0.974–2.491)	0.064	−0.15	0.884	NS
	<1 month	3	337	82	Fixed	0	0.749	0.838 (0.363–1.936)	0.680			NS
	<3 months	4	587	170	Random	62.8	0.045	2.229 (0.862–5.763)	0.098			NS
	Periungual erythema	6	853	229	Fixed	42.9	0.119	1.362 (0.861–2.155)	0.187	−1.78	0.150	NS
	<1 month	3	337	82	Fixed	0	0.706	1.261 (0.661–2.408)	0.482			NS
	<3 months	3	516	147	Random	74.8	0.019	1.019 (0.232–4.471)	0.980			NS
Clinical characteristics	Dyspnea	3	266	98	Random	91.7	<0.001	1.991 (0.533-7.441)	0.306	3.70	0.168	NS
	<1 month	1	103	44	NA	NA	NA	8.662 (2.923-25.665)	<0.001			RF
	<3 months	2	117	31	Random	80.4	0.024	1.013 (0.421–2.436)	0.977			NS
	Mechanic’s hands	8	1,275	352	Fixed	28.3	0.203	1.421 (1.054–1.915)	0.021	0.07	0.950	RF
	<1 month	3	337	82	Fixed	0	0.967	1.339 (0.735–2.441)	0.341			NS
	<3 months	5	938	270	Random	58.5	0.047	1.506 (0.826–2.747)	0.181			NS
	Fever	4	560	157	Fixed	50.2	0.110	3.090 (1.933–4.939)	<0.001	0.71	0.549	RF
	<1 month	0										NA
	<3 months	4	560	157	Fixed	50.2	0.110	3.090 (1.933–4.939)	<0.001	0.71	0.549	RF
	Dysphagia	4	623	154	Fixed	0	0.915	0.773 (0.653–0.916)	0.003	−0.39	0.737	PF
	<1 month	1	151	29	NA	NA	NA	0.588 (0.162–2.134)	0.419			NS
	<3 months	3	472	125	Fixed	0	0.842	0.777 (0.655–0.922)	0.004			PF
	V sign	5	569	181	Fixed	0	0.973	1.002 (0.647–1.552)	0.992	−0.75	0.508	NS
	<1 month	2	80	41	Fixed	0	0.583	0.866 (0.370–2.026)	0.741			NS
	<3 months	3	489	140	Fixed	0	0.976	1.056 (0.634–1.759)	0.833			NS
	Muscle weakness	6	809	207	Fixed	46.5	0.096	0.935 (0.623–1.403)	0.746	−0.20	0.851	NS
	<1 month	3	337	82	Fixed	52.1	0.124	1.400 (0.667–2.937)	0.374			NS
	<3 months	3	472	125	Fixed	43.7	0.169	0.787 (0.485–1.278)	0.333			NS
	Arthritis	7	652	196	Fixed	0	0.940	1.065 (0.740–1.533)	0.735	−0.23	0.829	NS
	<1 month	4	387	113	Fixed	0	0.713	1.048 (0.643–1.710)	0.850			NS
	<3 months	3	265	83	Fixed	0	0.825	1.086 (0.629–1.876)	0.767			NS
	Pulmonary infection	3	256	60	Fixed	0	0.616	2.610 (1.457–4.677)	0.001	0.37	0.736	RF
	<1 month	2	212	50	Fixed	0	0.638	2.313 (1.230–4.347)	0.009			RF
	<3 months	1	44	10	NA	NA	NA	5.303 (1.152-24.416)	0.032			RF
IIM subtypes	DM	3	538	136	Fixed	0	0.375	0.844 (0.534–1.335)	0.469	−0.68	0.619	NS
	<1 month	2	212	50	Fixed	0	0.601	0.618 (0.320–1.192)	0.151			NS
	<3 months	1	326	86	NA	NA	NA	1.134 (0.599–2.147)	0.699			NS
	CADM	3	241	65	Fixed	0	0.806	3.024 (1.491–6.130)	0.002	−1.26	0.426	RF
	<1 month	3	241	65	Fixed	0	0.806	3.024 (1.491–6.130)	0.002	−1.26	0.426	RF
	<3 months	0										RF
Antibody positivity	Anti-MDA5	10	1,143	292	Fixed	22.6	0.235	6.044 (4.331–8.435)	<0.001	1.00	0.347	RF
	<1 month	4	519	122	Fixed	50.5	0.109	6.262 (3.914-10.019)	<0.001			RF
	<3 months	6	624	170	Fixed	9.5	0.355	5.831 (3.635-9.355)	<0.001			RF
	Anti-Ro-52	8	1,298	331	Fixed	37.9	0.127	2.425 (1.807–3.255)	<0.001	0.61	0.564	RF
	<1 month	3	469	91	Random	65.6	0.055	2.689 (1.126–6.423)	0.026			RF
	<3 months	5	829	240	Fixed	21.0	0.281	2.264 (1.573–3.259)	<0.001			RF
	Anti-Jo-1	3	538	136	Fixed	0	0.685	0.650 (0.349–1.210)	0.174	72.38	0.009	NS
	<1 month	2	212	50	Fixed	0	0.605	0.852 (0.320–2.273)	0.750			NS
	<3 months	1	326	86	NA	NA	NA	0.542 (0.243–1.209)	0.135			NS
	Anti-TIF1-γ	3	538	136	Fixed	0	0.864	0.526 (0.194–1.427)	0.207	0.27	0.833	NS
	<1 month	2	212	50	Fixed	0	0.947	0.659 (0.181–2.404)	0.528			NS
	<3 months	1	326	86	NA	NA	NA	0.378 (0.079–1.810)	0.223			NS
	Anti-ARS	4	735	187	Random	69.2	0.021	0.742 (0.316–1.741)	0.493	−0.62	0.596	NS
	<1 month	2	307	72	Fixed	26.8	0.242	0.346 (0.133–0.904)	0.030			NS
	<3 months	2	428	115	Fixed	48.0	0.166	1.274 (0.589–2.757)	0.538			NS
	Anti-Ku	3	538	136	Fixed	0	0.841	1.099 (0.316–3.821)	0.883	0.47	0.719	NS
	<1 month	2	212	50	Fixed	0	0.566	1.012 (0.182–5.613)	0.989			NS
	<3 months	1	326	86	NA	NA	NA	1.205 (0.196-7.415)	0.841			NS
Laboratory data	Ferritin (>336 ng/ml)	9	794	287	Fixed	34	0.146	5.844 (4.121–8.287)	<0.001	0.4	0.7	RF
	<1 month	4	211	107	Random	68.6	0.023	4.737 (1.680-13.355)	0.003			RF
	<3 months	5	583	180	Fixed	0	0.714	6.407 (4.116-9.972)	<0.001			RF
	CK (≥47.5 U/L)	3	712	207	Random	69.7	0.037	0.994 (0.393–2.513)	0.989	−0.71	0.608	NS
	<1 month	1	39	20	NA	NA	NA	0.593 (0.088–4.003)	0.592			NS
	<3 months	2	673	187	Random	83.4	0.014	1.091 (0.356–3.349)	0.878			NS
	CRP (>0.05 mg/L)	5	833	253	Fixed	24.9	0.225	3.366 (2.149–5.274)	<0.001	2.85	0.065	RF
	<1 month	2	120	55	Fixed	0	0.688	4.771 (2.195-10.370)	<0.001			RF
	<3 months	3	713	198	Fixed	46.0	0.157	3.114 (1.666–5.823)	<0.001			RF
	LDH (>280 U/L)	4	785	230	Fixed	39.3	0.176	3.627 (2.406–5.466)	<0.001	−0.69	0.562	RF
	<1 month	0										NA
	<3 months	4	785	230	Fixed	39.3	0.176	3.627 (2.406–5.466)	<0.001	−0.69	0.562	RF
	ESR (>21 mm/h)	4	753	228	Fixed	16.3	0.310	1.598 (1.089–2.344)	0.016	0.43	0.707	RF
	<1 month	2	80	41	Fixed	37.7	0.205	2.523 (0.739–8.617)	0.140			NS
	<3 months	2	673	187	Fixed	28.1	0.238	1.521 (1.016–2.277)	0.041			RF
	AST (>36 U/L)	4	785	230	Fixed	0	0.513	2.666 (1.864–3.814)	<0.001	−0.04	0.971	RF
	<1 month	0										NA
	<3 months	4	785	230	Fixed	0	0.513	2.666 (1.864–3.814)	<0.001	−0.04	0.971	RF
	ALT (>27.75 U/L)	3	746	208	Fixed	27.2	0.253	2.702 (1.737–4.201)	<0.001	0.86	0.546	RF
	<1 month	0										NA
	<3 months	3	746	208	Fixed	27.2	0.253	2.702 (1.737–4.201)	<0.001	0.86	0.546	RF

p-Values were two-sided, and values of <0.05 were considered statistically signiﬁcant.

OR, odds ratios; CI, confidence interval; NA, not available; NS, no association; PF, protective factor; RF, risk factor; IIM, idiopathic inflammatory myopathy; RP-ILD, rapidly progressive interstitial lung disease; DM, dermatomyositis; CADM, clinically amyopathic dermatomyositis; MDA5, melanoma differentiation-associated 5; TIF-1γ, translation initiation factor-1γ; CK, creatine kinase; CRP, C-reactive protein; LDH, lactate dehydrogenase; ESR, erythrocyte sedimentation rate; AST, aspartate aminotransferase; ALT, alanine transaminase.

## Discussion

Clarifying the associated factors for RP-ILD development in IIM patients helps us gain a more systematic and comprehensive understanding of the disease, aiding in its intervention and management. Through pooled analysis of potential associated factors, we identified demographic characteristic (age), IIM subtype (CADM), clinical manifestations (mechanic’s hands, fever, dysphagia, and pulmonary infection), antibody activities (anti-MDA5 antibody and anti-Ro-52 antibody), and laboratory data (ferritin >336 ng/mL, LDH > 280 U/L, ESR > 21 mm/h, CRP > 0.05 mg/L, AST >36 U/L, and ALT >27.75 U/L) as associated risk factors for RP-ILD development, while longer disease duration and dysphagia were potential protective factors.

Pooled results showed that older age was an associated risk factor for RP-ILD development in IIM patients. A study by Zhang Y et al. (7) showed that the mean age at the onset of RP-ILD in IIM patients was greater than 50 years, which may be because aging of the lungs increases the probability of the occurrence of ILD (32). However, subgroup analysis showed that age had no significant correlation with RP-ILD development in IIM patients, whether in the subgroups with progression time <1 or <3 months. This result suggests that the impact of age on the risk of RP-ILD may be related to the rate of disease progression: elderly patients may be more likely to accumulate lung damage during chronic progression rather than directly drive rapid progression. Further studies are needed in the future to explore this association in greater detail.

A study by Li et al. (29) showed that a longer duration of the disease acted as a potential protective factor against RP-ILD development in IIM patients. Patients with longer disease durations inherently represent survivors who may have passed the highest-risk window for *de novo* fulminant lung injury. Moreover, a longer disease course may require a more stable ILD phenotype less prone to rapid progression. Additionally, patients with longer-standing disease are more likely to have received prolonged corticosteroid and immunosuppressive therapy, which may mitigate pulmonary inflammation and prevent the onset or acceleration of fibrosis. Thus, the observed protection may mainly reflect epidemiological reality rather than biological causation—those with longer courses had already cleared the danger zone before RP-ILD could develop.

Shorter disease durations suggest that RP-ILD occurs more often in the early stage of IIM, which may be similar to the mechanism by which coronavirus disease 2019 (COVID-19) leads to acute lung injury (33). It has been reported that patients with RP-ILD tend to exhibit a higher inflammatory load (34), especially in the early stage of the disease when they are in a state of systemic hyperinflammation. Inflammatory factors may directly affect the lung’s interstitial tissue, causing acute lung injury and the subsequent development of RP-ILD (33, 35). Fever in IIM may indicate systemic inflammatory activity or infection, which can lead to an elevated ESR. Similarly, elevated CRP and LDH represent systemic hyperinflammatory conditions, and all four serve as correlates of RP-ILD development in IIM patients (6, 28), which aligns with our summarized conclusions. Shi et al. (29) found that serum markers, such as CRP and LDH, combined with the level of elevated serum B-cell activating factor (BAFF), reflect the severity of lung injury and contribute to the early identification of RP-ILD in IIM patients.

Our pooled analysis identified mechanic’s hands as an associated risk factor for RP-ILD. This trend was consistent in subgroup analyses, although without statistical significance, potentially due to the limited number of studies included in these subgroups. Pulmonary infection was an associated risk factor in both subgroups for RP-ILD development, and the mechanism of action is complex, potentially involving multiple levels of immunosuppressive therapy, structural lung damage, and a vicious cycle of infection and inflammation (36).

Elevated serum ferritin and anti-MDA5 antibody are the strongest risk factors for developing RP-ILD in IIM patients, as reported in the present study, which is consistent with the findings of He et al. (37) and Xu et al. (21). MDA5 is a viral cytoplasmic sensor involved in the innate immune response, recognizing viral RNA and activating the expression of type I interferon (38). In susceptible individuals, anti-MDA5 antibodies generated during the autoimmune process amplify the type I interferon (IFN) response. This provokes a hyperinflammatory state characterized by a cytokine storm—driven by an antiviral, pro-inflammatory network orchestrated by activated monocytes and macrophages—that directly damages the pulmonary vascular endothelium (39, 40). Serum ferritin levels are a crucial biomarker for macrophage activation syndrome and a predictor of RP-ILD (41, 42). Ferritin, a key molecule for storing iron, is secreted by activated macrophages and plays an essential role in sequestering potentially harmful reactive iron molecules. High serum ferritin levels in patients with RP-ILD may, through macrophage activation, indicate abnormal ferritin level production (21). Based on the first clustering analysis in a large sample of anti-MDA5^+^DM patients in Asia, which revealed the characteristics of RP-ILD in high-inflammatory state anti-MDA5^+^DM patients, the coexistence of anti-Ro-52 antibodies and anti-MDA5 antibodies predicted high-risk RP-ILD patients (43). Like the anti-MDA5 antibody, the anti-Ro-52 antibody is a specific biomarker for IIM-associated RP-ILD (6, 19) and is strongly associated with the development of RP-ILD.

Liver dysfunction in DM patients was linked to anti-MDA5, which was significantly associated with RP-ILD (44). AST (>36 U/L) and ALT (>27.75 U/L) were associated factors for RP-ILD development in IIM patients, which aligns with the findings of Li et al. (27). The mechanism may involve activating hepatic Kupffer cells and other macrophages, leading to liver injury. Therefore, ALT and AST levels are elevated (45), and alveolar macrophages are activated, leading to neutrophil activation and subsequent pulmonary fibrosis (25).

Our pooled results indicate that IIM patients with dysphagia are associated with a lower risk of developing RP-ILD, which may be related to the following factors: first, different myositis-specific autoantibodies (MSAs) are associated with different clinical phenotypes in IIM (46). Previous studies have found that MSAs (such as anti-TIF1-γ and anti-HMGCR) associated with dysphagia in IIM patients are often accompanied by a lower risk of ILD (46, 47). Second, some MSAs may be more likely to involve the muscle tissue in IIM patients (48) [anti-Jo-1 antibodies (49)] and less likely to involve the lungs. Third, patients with IIM who have dysphagia are more likely to receive intravenous corticosteroid therapy (50), which may serve as a potential factor in reducing the incidence of RP-ILD in IIM patients. Last, patients with CADM, as a type of IIM, were less likely to have dysphagia compared to typical DM (51, 52). The presence of dysphagia symptoms in IIM patients can serve as an important clue for non-CADM patients and is also associated with a lower risk of RP-ILD. However, the specific mechanisms underlying the association between dysphagia and a lower risk of RP-ILD remain unclear, and further research is needed to explore this topic in the future.

There are some limitations in our pooled study: i) all included studies were small-sample and retrospective study designs, and the associated factors obtained from the pooled results need to be further clarified by large-sample, prospective, multicenter studies. ii) We did not perform pooled analyses of the potential risk factors [such as Antinuclear antibody (ANA) (6, 20, 28), HRCT pattern (6, 7), and Krebs Von den Lungen-6 (KL-6) (14, 24, 26, 31)] and outcomes of RP-ILD associated with recurrence, hospitalization, and mortality in some RP-ILD patients. This was primarily due to insufficient evidence for meta-analysis or the inability to harmonize the units of classification variables. iii) Some of the included studies did not perform stratified analyses for antibody subtypes or ILD phenotypes (e.g., histopathologic subtypes). iv) Most of the studies originated from the Asian region, which may limit the generalizability of the findings. v) Future studies should conduct meta-analyses with sufficient MSA data to further evaluate valuable antibodies for predicting RP-ILD development in IIM patients. vi) Due to the lack of studies on the thresholds for factors associated with the development of RP-ILD in IIM patients, our study is merely an exploratory attempt to identify relevant parameter thresholds. The thresholds we have identified should be interpreted with caution in clinical practice, and we suggest that large-scale prospective studies be conducted in the future to verify the reliability of these thresholds.

## Conclusion

In conclusion, our pooled analyses showed that advanced age, CADM, mechanic’s hands, fever, dysphagia, pulmonary infection, anti-MDA5 antibody, anti-Ro-52 antibody, ferritin (>336 ng/mL), LDH (>280 U/L), ESR (>21 mm/h), CRP (>0.05 mg/L), AST (>36 U/L), and ALT (>27.75 U/L) were risk factors for RP-ILD development in IIM patients. In contrast, longer disease duration and dysphagia were protective factors against RP-ILD development, potentially providing a key decision node for the early identification of high-risk patients and providing an essential basis for clinical risk stratification and intervention decisions.

## Data Availability

The original contributions presented in the study are included in the article/**Supplementary Material**. Further inquiries can be directed to the corresponding author.

## Supplementary material

Supplementary Table 1 Database and Search strategy.

Supplementary Table 2 Definitions of Rapidly Progressive Interstitial Lung Disease (RP-ILD) in Included Studies.

Supplementary Table 3 Results of Newcastle-Ottawa quality assessment Scale for each included study.

Supplementary Figure 1 Plot for the assessment of heterogeneity among the included studies of demographic characteristics [**(A)** Female, **(B)** Disease duration, **(C)** Age] of RP-ILD development in IIM patients through one-by-one elimination method.

Supplementary Figure 2 Plot for the assessment of heterogeneity among the included studies of skin manifestations [**(A)** Gottron papules, **(B)** Heliotrope rash, **(C)**. Shawl sign, **(D)** Skin ulceration, **(E)** Periungual erythema] of RP-ILD development in IIM patients through one-by-one elimination method.

Supplementary Figure 3 Plot for the assessment of heterogeneity among the included studies of clinical characteristics [**(A)** Dyspnea, **(B)**. Mechanic's hands, **(C)** Fever, **(D)** Dysphagia, **(E)** V sign, **(F)** Muscle weakness, **(G)** Arthritis, **(H)** Pulmonary infection] of RP-ILD development in IIM patients through one-by-one elimination method.

Supplementary Figure 4 Plot for the assessment of heterogeneity among the included studies of IIM subtypes [**(A)** DM, **(B)** CADM] of RP-ILD development in IIM patients through one-by-one elimination method.

Supplementary Figure 5 Plot for the assessment of heterogeneity among the included studies of antibody activity [**(A)** Anti-MDA5, **(B)** Anti-Ro-52, **(C)** Anti-Jo-1, **(D)** Anti-TIF1-γ, **(E)** Anti-ARS, **(D)** Anti-Ku] of RP-ILD development in IIM patients through one-by-one elimination method.

Supplementary Figure 6 Plot for the assessment of heterogeneity among the included studies of serum biomarkers [**(A)** Ferritin(>336ng/ml), **(B)** CK(≥47.5U/L), **(C)** CRP(>0.05mg/L), **(D)** LDH(>280U/L), **(E)** ESR(>21mm/h), **(F)** AST(>36U/L), **(G)** ALT(>27.75U/L)] of RP-ILD development in IIM patients through one-by-one elimination method.

Supplementary Figure 7 Plot for the assessment of heterogeneity among the included studies of serum biomarkers [**(A)** Ferritin(>336ng/ml), **(B)** CK(≥47.5U/L), **(C)** CRP(>0.05mg/L), **(D)** LDH(>280U/L), **(E)** ESR(>21mm/h), **(F)** AST(>36U/L), **(G)**. ALT(>27.75U/L)] of RP-ILD development in IIM patients through one-by-one elimination method.

Supplementary Figure 8 Funnel plots of trim-and-fill analysis for odds ratio of RP-ILD development in IIM patients [**(A)** Gottron papules, **(B)** Heliotrope rash, **(C)** Shawl sign, **(D)** Skin ulceration, **(E)** Periungual erythema].

Supplementary Figure 9 Funnel plots of trim-and-fill analysis for odds ratio of RP-ILD development in IIM patients [**(A)** Dyspnea, **(B)** Mechanic's hands, **(C)** Fever, **(D)** Dysphagia, **(E)** V sign, **(F)** Muscle weakness, **(G)** Arthritis, **(H)** Pulmonary infection].

Supplementary Figure 10 Funnel plots of trim-and-fill analysis for odds ratio of RP-ILD development in IIM patients [**(A)** DM, **(B)** CADM].

Supplementary Figure 11 Funnel plots of trim-and-fill analysis for odds ratio of RP-ILD development in IIM patients [**(A)** Anti-MDA5, **(B)** Anti-Ro-52, **(C)** Anti-Jo-1, **(D)** Anti-TIF1-γ, **(E)** Anti-ARS, **(F)** Anti-Ku].

Supplementary Figure 12 Funnel plots of trim-and-fill analysis for odds ratio of RP-ILD development in IIM patients [**(A)** Ferritin(>336ng/ml), **(B)** CK(≥47.7U/L), **(C)** CRP(>0.05mg/L), **(D)** LDH(>280U/L), **(E)**. ESR(>21mm/h), **(F)** AST(>36U/L), **(G)** ALT(>27.75U/L)].

## References

[1] Findlay AR Goyal NA and Mozaffar T . An overview of polymyositis and dermatomyositis. Muscle nerve. (2015) 51:638–56. doi: 10.1002/mus.24566, PMID: 25641317

[2] Fathi M Lundberg IE and Tornling G . Pulmonary complications of polymyositis and dermatomyositis. Semin Respir Crit Care Med. (2007) 28:451–8. doi: 10.1055/s-2007-985666, PMID: 17764062

[3] Fathi M Lundberg IE . Interstitial lung disease in polymyositis and dermatomyositis. Curr Opin Rheumatol. (2005) 17:701–6. doi: 10.1097/01.bor.0000179949.65895.53, PMID: 16224246

[4] Ye S Chen XX Lu XY Wu MF Deng Y Huang WQ . Adult clinically amyopathic dermatomyositis with rapid progressive interstitial lung disease: a retrospective cohort study. Clin Rheumatol. (2007) 26:1647–54. doi: 10.1007/s10067-007-0562-9, PMID: 17308858

[5] Wu W Guo L Fu Y Wang K Zhang D Xu W . Interstitial lung disease in anti-MDA5 positive dermatomyositis. Clin Rev Allergy Immunol. (2021) 60:293–304. doi: 10.1007/s12016-020-08822-5, PMID: 33405101

[6] Li Y Li Y Wang Y Shi L Lin F Zhang Z . A clinical risk model to predict rapidly progressive interstitial lung disease incidence in dermatomyositis. Front Med (Lausanne). (2021) 8:733599. doi: 10.3389/fmed.2021.733599, PMID: 34646845 PMC8502922

[7] Zhang Y Ge Y Yang H Chen H Tian X Huang Z . Clinical features and outcomes of the patients with anti-glycyl tRNA synthetase syndrome. Clin Rheumatol. (2020) 39:2417–24. doi: 10.1007/s10067-020-04979-8, PMID: 32144624

[8] Page MJ McKenzie JE Bossuyt PM Boutron I Hoffmann TC Mulrow CD . The PRISMA 2020 statement: an updated guideline for reporting systematic reviews. BMJ (Clinical Res ed). (2021) 372:n71. doi: 10.1136/bmj.n71, PMID: 33782057 PMC8005924

[9] Lundberg IE Tjärnlund A Bottai M Werth VP Pilkington C de Visser M . European league against rheumatism/American college of rheumatology classification criteria for adult and juvenile idiopathic inflammatory myopathies and their major subgroups. Arthritis Rheumatol (Hoboken NJ). (2017) 69:2271–82. doi: 10.1002/art.40320, PMID: 29106061 PMC5846474

[10] Bohan A Peter JB . Polymyositis and dermatomyositis (first of two parts). New Engl J Med. (1975) 292:344–7. doi: 10.1056/nejm197502132920706, PMID: 1090839

[11] Cavagna L Trallero-Araguás E Meloni F Cavazzana I Rojas-Serrano J Feist E . Influence of antisynthetase antibodies specificities on antisynthetase syndrome clinical spectrum time course. J Clin Med. (2019) 8(11):2013. doi: 10.3390/jcm8112013, PMID: 31752231 PMC6912490

[12] Stang A . Critical evaluation of the Newcastle-Ottawa scale for the assessment of the quality of nonrandomized studies in meta-analyses. Eur J Epidemiol. (2010) 25:603–5. doi: 10.1007/s10654-010-9491-z, PMID: 20652370

[13] He W Cui B Chu Z Chen X Liu J Pang X . Radiomics based on HRCT can predict RP-ILD and mortality in anti-MDA5 + dermatomyositis patients: a multi-center retrospective study. Respir Res. (2024) 25:252. doi: 10.1186/s12931-024-02843-w, PMID: 38902680 PMC11191144

[14] Li C Han Y Li X Zhang H Yao Z Zhou J . Soluble CXCL16 is a prognostic biomarker associated with rapidly progressive interstitial lung disease complicated with dermatomyositis. Semin Arthritis rheumatism. (2024) 67:152483. doi: 10.1016/j.semarthrit.2024.152483, PMID: 38843569

[15] Liang J Cao H Liu Y Ye B Sun Y Ke Y . The lungs were on fire: a pilot study of (18)F-FDG PET/CT in idiopathic-inflammatory-myopathy-related interstitial lung disease. Arthritis Res Ther. (2021) 23:198. doi: 10.1186/s13075-021-02578-9, PMID: 34301306 PMC8298695

[16] Liang J Cao H Yang Y Ke Y Yu Y Sun C . Efficacy and tolerability of nintedanib in idiopathic-inflammatory-myopathy-related interstitial lung disease: A pilot study. Front Med (Lausanne). (2021) 8:626953. doi: 10.3389/fmed.2021.626953, PMID: 33614683 PMC7886679

[17] Shen YW Zhang YM Huang ZG Wang GC and Peng QL . Increased levels of soluble CD206 associated with rapidly progressive interstitial lung disease in patients with dermatomyositis. Mediators Inflamm. (2020) 2020:7948095. doi: 10.1155/2020/7948095, PMID: 33192174 PMC7641712

[18] Shi Y You H Liu C Qiu Y Lv C Zhu Y . Elevated serum B-cell activator factor levels predict rapid progressive interstitial lung disease in anti-melanoma differentiation associated protein 5 antibody positive dermatomyositis. Orphanet J Rare Dis. (2024) 19:170. doi: 10.1186/s13023-024-03153-6, PMID: 38637830 PMC11027411

[19] Tsai HC Chen WS Sun YS Lai CC Yang YY Chou WR . Antibodies against Small Ubiquitin-like Modifier Activating Enzyme May Be a Protective Factor from Rapid Progressive Interstitial Lung Disease in Patients Bearing Antibodies against Melanoma Differentiation Associated Gene 5. J Clin Med. (2024) 13(3):725. doi: 10.3390/jcm13030725, PMID: 38337419 PMC10856636

[20] Wang K Tian Y Liu S Zhang Z Shen L Meng D . Risk factors and predictive model for dermatomyositis associated with rapidly progressive interstitial lung disease. Pharmgenomics Pers Med. (2022) 15:775–83. doi: 10.2147/pgpm.S369556, PMID: 36071824 PMC9444234

[21] Xu Y Yang CS Li YJ Liu XD Wang JN Zhao Q . Predictive factors of rapidly progressive-interstitial lung disease in patients with clinically amyopathic dermatomyositis. Clin Rheumatol. (2016) 35:113–6. doi: 10.1007/s10067-015-3139-z, PMID: 26660480

[22] Zhang H Liang R Yuan X Zheng Z and Lai W . Serum IgA levels for predicting the development of rapidly progressive interstitial lung disease in dermatomyositis. Respir Med. (2023) 216:107322. doi: 10.1016/j.rmed.2023.107322, PMID: 37302423

[23] Zhang Y Chen Z Long Y Zhang B He Q Tang K . 18F-FDG PET/CT and HRCT: a combined tool for risk stratification in idiopathic inflammatory myopathy-associated interstitial lung disease. Clin Rheumatol. (2022) 41:3095–105. doi: 10.1007/s10067-022-06239-3, PMID: 35759126

[24] Zhu Y Wang L Sun Y Wang J Lv C You H . Serum Krebs von den Lungen-6 concentrations reflect severity of anti-melanoma differentiation-associated protein 5 antibody positive dermatomyositis associated interstitial lung disease. Clin Exp Rheumatol. (2022) 40:292–7. doi: 10.55563/clinexprheumatol/zmn18h, PMID: 34874831

[25] Zhu D Qiao J Tang S Pan Y Li S Yang C . Elevated carcinoembryonic antigen predicts rapidly progressive interstitial lung disease in clinically amyopathic dermatomyositis. Rheumatol (Oxford England). (2021) 60:3896–903. doi: 10.1093/rheumatology/keaa819, PMID: 33398346

[26] Karino K Kono M Kono M Sakamoto K Fujieda Y Kato M . Myofascia-dominant involvement on whole-body MRI as a risk factor for rapidly progressive interstitial lung disease in dermatomyositis. Rheumatol (Oxford England). (2020) 59:1734–42. doi: 10.1093/rheumatology/kez642, PMID: 31925431

[27] Li M Zhao X Liu B Zhao Y Li X Ma Z . Predictors of rapidly progressive interstitial lung disease and prognosis in Chinese patients with anti-melanoma differentiation-associated gene 5-positive dermatomyositis. Front Immunol. (2023) 14:1209282. doi: 10.3389/fimmu.2023.1209282, PMID: 37691917 PMC10483132

[28] Guo L Zhang X Pu W Zhao J Wang K Zhang D . WDFY4 polymorphisms in Chinese patients with anti-MDA5 dermatomyositis is associated with rapid progressive interstitial lung disease. Rheumatol (Oxford England). (2023) 62:2320–4. doi: 10.1093/rheumatology/kead006, PMID: 36637178

[29] Li Y Deng W Zhou Y Luo Y Wu Y Wen J . A nomogram based on clinical factors and CT radiomics for predicting anti-MDA5+ DM complicated by RP-ILD. Rheumatol (Oxford England). (2024) 63:809–16. doi: 10.1093/rheumatology/kead263, PMID: 37267146

[30] Sagawa T Kida T Inaba T Yokota I Sagawa R Kasahara A . Utility of coagulation markers for the prediction of rapidly progressive interstitial lung disease in patients with dermatomyositis. Lung. (2019) 197:437–42. doi: 10.1007/s00408-019-00245-0, PMID: 31240390

[31] Yang Q Li T Zhang X Lyu K Wu S Chen Y . Initial predictors for short-term prognosis in anti-melanoma differentiation-associated protein-5 positive patients. Orphanet J Rare Dis. (2021) 16:58. doi: 10.1186/s13023-021-01705-8, PMID: 33516242 PMC7847582

[32] Cho SJ Stout-Delgado HW . Aging and lung disease. Annu Rev Physiol. (2020) 82:433–59. doi: 10.1146/annurev-physiol-021119-034610, PMID: 31730381 PMC7998901

[33] Ciesla DJ Moore EE Johnson JL Cothren CC Banerjee A Burch JM . Decreased progression of postinjury lung dysfunction to the acute respiratory distress syndrome and multiple organ failure. Surgery. (2006) 140:640–7. doi: 10.1016/j.surg.2006.06.015, PMID: 17011912

[34] Matsuda S Kotani T Ishida T Fukui K Fujiki Y Suzuka T . Exploration of pathomechanism using comprehensive analysis of serum cytokines in polymyositis/dermatomyositis-interstitial lung disease. Rheumatol (Oxford England). (2020) 59:310–8. doi: 10.1093/rheumatology/kez301, PMID: 31321420

[35] Zheng Z Peng F and Zhou Y . Pulmonary fibrosis: A short- or long-term sequelae of severe COVID-19? Chin Med J pulmonary Crit Care Med. (2023) 1:77–83. doi: 10.1016/j.pccm.2022.12.002, PMID: 37388822 PMC9988550

[36] Xiao Y Zuo X You Y Luo H Duan L Zhang W . Investigation into the cause of mortality in 49 cases of idiopathic inflammatory myopathy: A single center study. Exp Ther Med. (2016) 11:885–9. doi: 10.3892/etm.2016.3006, PMID: 26998007 PMC4774336

[37] He X Ji J Chen X Luo Z Fang S Yan H . Serum ferritin as a significant biomarker for patients with idiopathic inflammatory myopathy-associated interstitial lung disease: A systematic review and meta-analysis. Semin Arthritis rheumatism. (2024) 64:152350. doi: 10.1016/j.semarthrit.2023.152350, PMID: 38086199

[38] Sato S Hoshino K Satoh T Fujita T Kawakami Y Fujita T . RNA helicase encoded by melanoma differentiation-associated gene 5 is a major autoantigen in patients with clinically amyopathic dermatomyositis: Association with rapidly progressive interstitial lung disease. Arthritis rheumatism. (2009) 60:2193–200. doi: 10.1002/art.24621, PMID: 19565506

[39] Gono T Okazaki Y and Kuwana M . Antiviral proinflammatory phenotype of monocytes in anti-MDA5 antibody-associated interstitial lung disease. Rheumatol (Oxford England). (2022) 61:806–14. doi: 10.1093/rheumatology/keab371, PMID: 33890985

[40] Mehta P MaChado PM and Gupta L . Understanding and managing anti-MDA 5 dermatomyositis, including potential COVID-19 mimicry. Rheumatol Int. (2021) 41:1021–36. doi: 10.1007/s00296-021-04819-1, PMID: 33774723 PMC8000693

[41] Zou J Guo Q Chi J Wu H and Bao C . HRCT score and serum ferritin level are factors associated to the 1-year mortality of acute interstitial lung disease in clinically amyopathic dermatomyositis patients. Clin Rheumatol. (2015) 34:707–14. doi: 10.1007/s10067-015-2866-5, PMID: 25609178

[42] Gono T Kawaguchi Y Hara M Masuda I Katsumata Y Shinozaki M . Increased ferritin predicts development and severity of acute interstitial lung disease as a complication of dermatomyositis. Rheumatol (Oxford England). (2010) 49:1354–60. doi: 10.1093/rheumatology/keq073, PMID: 20385617

[43] Xu L You H Wang L Lv C Yuan F Li J . Identification of three different phenotypes in anti-melanoma differentiation-associated gene 5 antibody-positive dermatomyositis patients: implications for prediction of rapidly progressive interstitial lung disease. Arthritis Rheumatol (Hoboken NJ). (2023) 75:609–19. doi: 10.1002/art.42308, PMID: 35849805

[44] Nagashima T Kamata Y Iwamoto M Okazaki H Fukushima N and Minota S . Liver dysfunction in anti-melanoma differentiation-associated gene 5 antibody-positive patients with dermatomyositis. Rheumatol Int. (2019) 39:901–9. doi: 10.1007/s00296-019-04255-2, PMID: 30790016

[45] Li M Yan S Dong R Xiang W Ma Z and Yang Q . Elevated platelet-to-lymphocyte ratio and neutrophil-to-lymphocyte ratio in patients with polymyositis/dermatomyositis: a retrospective study. Clin Rheumatol. (2023) 42:1615–24. doi: 10.1007/s10067-023-06542-7, PMID: 36781682

[46] Li S Ge Y Yang H Wang T Zheng X Peng Q . The spectrum and clinical significance of myositis-specific autoantibodies in Chinese patients with idiopathic inflammatory myopathies. Clin Rheumatol. (2019) 38:2171–9. doi: 10.1007/s10067-019-04503-7, PMID: 30863950

[47] Betteridge Z McHugh N . Myositis-specific autoantibodies: an important tool to support diagnosis of myositis. J Internal Med. (2016) 280:8–23. doi: 10.1111/joim.12451, PMID: 26602539

[48] Sun KY Fan Y Wang YX Zhong YJ and Wang GF . Prevalence of interstitial lung disease in polymyositis and dermatomyositis: A meta-analysis from 2000 to 2020. Semin Arthritis rheumatism. (2021) 51:175–91. doi: 10.1016/j.semarthrit.2020.11.009, PMID: 33383294

[49] Li JC Lai ZH Shao M Jin YB Gao XJ Zhang K . Significance of anti-Jo-1 antibody’s clinical stratification in idiopathic inflammatory myopathy and disease spectrum. Beijing da xue xue bao Yi xue ban = J Peking Univ Health Sci. (2023) 55:958–65. doi: 10.19723/j.issn.1671-167X.2023.06.002, PMID: 38101775 PMC10723990

[50] Birch C Tikly M and Govind N . Clinical spectrum and outcomes of idiopathic inflammatory myopathies in South Africans. Front Med (Lausanne). (2023) 10:1097824. doi: 10.3389/fmed.2023.1097824, PMID: 36860335 PMC9968836

[51] Pandya R Kleitsch J Lim D and Werth VP . Clinical characteristics and symptom progression of dermatomyositis subtypes: A retrospective analysis of a prospective database. J Am Acad Dermatol. (2024) 91:31–6. doi: 10.1016/j.jaad.2024.02.007, PMID: 38342246 PMC12317777

[52] Sasegbon A Hamdy S . The anatomy and physiology of normal and abnormal swallowing in oropharyngeal dysphagia. Neurogastroenterol motility. (2017) 29. doi: 10.1111/nmo.13100, PMID: 28547793

