# Supplementary material for: Associated factors in the development of rapidly progressive interstitial lung disease in patients with idiopathic inflammatory myopathies: a systematic review and meta-analysis
Source: Front Immunol. 2025 Aug 5;16:1628928. doi: 10.3389/fimmu.2025.1628928 (PMC12361146; doi:10.3389/fimmu.2025.1628928)
Supplement: Supplementary Table 1 — Database and Search strategy. [file DataSheet1.zip › Supplementary Material.docx]

Supplementary Table 1. Database and Search strategy

| **Database** | **Search strategy** |
| --- | --- |
| **Pubmed**  **(n=471)** | #1: (((RP-ILD[Title/Abstract]) OR (rapidly progressive interstitial lung disease[Title/Abstract])) OR (rapidly progressing interstitial lung disease[Title/Abstract])) OR (rapidly progressive ILD[Title/Abstract])  = 708 items |
|  | #2: (((((((((((((((((((((((((((((Myositis[MeSH Terms]) OR (Myositides[Title/Abstract])) OR (Inflammatory Muscle Diseases[Title/Abstract])) OR (Inflammatory Muscle Disease[Title/Abstract])) OR (Muscle Disease, Inflammatory[Title/Abstract])) OR (Inflammatory Myopathy[Title/Abstract])) OR (Inflammatory Myopathies[Title/Abstract])) OR (Myopathies, Inflammatory[Title/Abstract])) OR (Muscle Diseases, Inflammatory[Title/Abstract])) OR (Myopathy, Inflammatory[Title/Abstract])) OR (Myositis, Focal[Title/Abstract])) OR (Focal Myositides[Title/Abstract])) OR (Focal Myositis[Title/Abstract])) OR (Myositides, Focal[Title/Abstract])) OR (Myositis, Proliferative[Title/Abstract])) OR (Myositides, Proliferative[Title/Abstract])) OR (Proliferative Myositides[Title/Abstract])) OR (Proliferative Myositis[Title/Abstract])) OR (Myositis, Infectious[Title/Abstract])) OR (Infectious Myositides[Title/Abstract])) OR (Myositides, Infectious[Title/Abstract])) OR (Infectious Myositis[Title/Abstract])) OR (Idiopathic Inflammatory Myopathies[Title/Abstract])) OR (Myopathy, Idiopathic Inflammatory[Title/Abstract])) OR (Inflammatory Myopathies, Idiopathic[Title/Abstract])) OR (Myopathies, Idiopathic Inflammatory[Title/Abstract])) OR (Idiopathic Inflammatory Myopathy[Title/Abstract])) OR (Idiopathic Inflammatory Myositis[Title/Abstract])) OR (Inflammatory Myopathy, Idiopathic[Title/Abstract])) OR (IIM[Title/Abstract])  = 25684 items |
|  | #3: (((((((((((Dermatomyositis[MeSH Terms]) OR (Polymyositis-Dermatomyositis[Title/Abstract])) OR (Polymyositis Dermatomyositis[Title/Abstract])) OR (Dermatopolymyositis[Title/Abstract])) OR (Dermatomyositis, Adult Type[Title/Abstract])) OR (Adult Type Dermatomyositis[Title/Abstract])) OR (Dermatomyositis, Childhood Type[Title/Abstract])) OR (Childhood Type Dermatomyositis[Title/Abstract])) OR (Juvenile Dermatomyositis[Title/Abstract])) OR (Dermatomyositis, Juvenile[Title/Abstract])) OR (Juvenile Myositis[Title/Abstract])) OR (Myositis, Juvenile[Title/Abstract])  = 10370 items |
|  | #4: ((((((((((Polymyositis[MeSH Terms]) OR (Polymyositides[Title/Abstract])) OR (Myositis, Multiple[Title/Abstract])) OR (Multiple Myositis[Title/Abstract])) OR (Myositides, Multiple[Title/Abstract])) OR (Polymyositis Ossificans[Title/Abstract])) OR (Ossificans, Polymyositis[Title/Abstract])) OR (Polymyositis, Idiopathic[Title/Abstract])) OR (Idiopathic Polymyositides[Title/Abstract])) OR (Idiopathic Polymyositis[Title/Abstract])) OR (Polymyositides, Idiopathic[Title/Abstract])  = 12581 items |
|  | #5: ((((((((Myositis, Inclusion Body[MeSH Terms]) OR (Inclusion Body Myositides[Title/Abstract])) OR (Myositides, Inclusion Body[Title/Abstract])) OR (Inclusion Body Myositis[Title/Abstract])) OR (Inclusion Body Myopathy, Sporadic[Title/Abstract])) OR (Inclusion Body Myositis, Sporadic[Title/Abstract])) OR (Myositis, Inclusion Body, Sporadic[Title/Abstract])) OR (Sporadic Inclusion Body Myositis[Title/Abstract])) OR (Myopathy, Inclusion Body, Sporadic[Title/Abstract])  = 2398 items |
|  | #6: Amyopathic dermatomyositis[Title/Abstract]  = 617 items |
|  | #7: Antisynthetase syndrome[Title/Abstract]  = 654 items |
|  | #8: #2 OR #3 OR #4 OR #5 OR #6 OR #7= 27192 items |
|  | #9: #1 AND #8= **471** items |
| **Embase**  **(n=922)** | #1: 'rapidly progressive interstitial lung disease':ab,ti OR 'rapidly progressing interstitial lung disease':ab,ti OR 'rapidly progressive ILD':ab,ti OR 'RP-ILD':ab,ti  = 1397 items |
|  | #2: 'myositis'/exp OR 'allergic myositis':ab,ti OR 'inflammatory myopathy':ab,ti OR 'muscle inflammation':ab,ti OR 'myopathy, inflammatory':ab,ti OR 'myositis, allergic':ab,ti OR 'myositis':ab,ti  = 62377 items |
|  | #3: 'dermatomyositis'/exp OR 'dermato-myositis':ab,ti OR 'dermatomucomyositis':ab,ti OR 'dermatomyositides':ab,ti OR 'dermatopolymyositis':ab,ti OR 'polymyositis arthropathica':ab,ti OR 'unverricht-wagner syndrome':ab,ti OR 'wagner-unverricht syndrome':ab,ti OR 'wegner hepp unverrricht disease':ab,ti OR 'dermatomyositis':ab,ti  = 24052 items |
|  | #4: 'polymyositis'/exp OR 'inomyositis':ab,ti OR 'multiple myositis':ab,ti OR 'myositis, poly':ab,ti OR 'poly-myositis':ab,ti OR 'polymyositides':ab,ti OR 'polymyositis':ab,ti  = 13325 items |
|  | #5: 'inclusion body myositis'/exp OR 'inflammatory ibm':ab,ti OR 'myositis, inclusion body':ab,ti OR 'inclusion body myositis':ab,ti  = 4040 items |
|  | #6: 'amyopathic dermatomyositis'/exp OR ('amyopathic dm':ab,ti AND dermatomyositis:ab,ti) OR 'dermatomyositis sine myositis':ab,ti OR 'dermatomyositis without myositis':ab,ti OR 'amyopathic dermatomyositis':ab,ti  = 1335 items |
|  | #7: 'antisynthetase syndrome'/exp OR 'anti-jo1 syndrome':ab,ti OR 'anti-synthetase syndrome':ab,ti OR 'anti-synthetases syndrome':ab,ti OR 'antisynthetases syndrome':ab,ti OR 'antisynthetase syndrome':ab,ti  = 2264 items |
|  | #8: #2 OR #3 OR #4 OR #5 OR #6 OR #7  = 64021 items |
|  | #9: #1 AND #8  = **922** items |
| **Web of science**  **(n=577)** | #1: TS= (rapidly progressive interstitial lung disease OR rapidly progressing interstitial lung disease OR rapidly progressive ILD OR RP-ILD)  = 864 items |
|  | #2: TS= (Myositis OR Myositides OR Inflammatory Muscle Diseases OR Inflammatory Muscle Disease OR Muscle Disease, Inflammatory OR Inflammatory Myopathy OR Inflammatory Myopathies OR Myopathies, Inflammatory OR Muscle Diseases, Inflammatory OR Myopathy, Inflammatory OR Myositis, Focal OR Focal Myositides OR Focal Myositis OR Myositides, Focal OR Myositis, Proliferative OR Myositides, Proliferative OR Proliferative Myositides OR Proliferative Myositis OR Myositis, Infectious OR Infectious Myositides OR Myositides, Infectious OR Infectious Myositis OR Idiopathic Inflammatory Myopathies OR Myopathy, Idiopathic Inflammatory OR Inflammatory Myopathies, Idiopathic OR Myopathies, Idiopathic Inflammatory OR Idiopathic Inflammatory Myopathy OR Idiopathic Inflammatory Myositis OR Inflammatory Myopathy, Idiopathic OR IIM)  = 41419 items |
|  | #3: TS= (Dermatomyositis OR Polymyositis-Dermatomyositis OR Polymyositis Dermatomyositis OR Dermatopolymyositis OR Dermatomyositis, Adult Type OR Adult Type Dermatomyositis OR Dermatomyositis, Childhood Type OR Childhood Type Dermatomyositis OR Juvenile Dermatomyositis OR Dermatomyositis, Juvenile OR Juvenile Myositis OR Myositis, Juvenile)  = 13803 items |
|  | #4: TS= (Polymyositis OR Polymyositides OR Myositis, Multiple OR Multiple Myositis OR Myositides, Multiple OR Polymyositis Ossificans OR Ossificans, Polymyositis OR Polymyositis, Idiopathic OR Idiopathic Polymyositides OR Idiopathic Polymyositis OR Polymyositides, Idiopathic)  = 10067 items |
|  | #5: TS= (Myositis, Inclusion Body OR Inclusion Body Myositides OR Myositides, Inclusion Body OR Inclusion Body Myositis OR Inclusion Body Myopathy, Sporadic OR Inclusion Body Myositis, Sporadic OR Myositis, Inclusion Body, Sporadic OR Sporadic Inclusion Body Myositis OR Myopathy, Inclusion Body, Sporadic)  = 3414 items |
|  | #6: TS= (Amyopathic dermatomyositis)  = 1162 items |
|  | #7: TS= (Antisynthetase syndrome)  = 961 items |
|  | #8: #2 OR #3 OR #4 OR #5 OR #6 OR #7  =52171 items |
|  | #9: #1 AND #8  = **577** items |
| **Scopus**  **(n=485)** | #1: TITLE-ABS-KEY ("Myositis" OR "Myositides" OR "Inflammatory Muscle Diseases" OR "Inflammatory Muscle Disease" OR "Muscle Disease, Inflammatory" OR "Inflammatory Myopathy" OR "Inflammatory Myopathies" OR "Myopathies, Inflammatory" OR "Muscle Diseases, Inflammatory" OR "Myopathy, Inflammatory" OR "Myositis, Focal" OR "Focal Myositides" OR "Focal Myositis" OR "Myositides, Focal" OR "Myositis, Proliferative" OR "Myositides, Proliferative" OR "Proliferative Myositides" OR "Proliferative Myositis" OR "Myositis, Infectious" OR "Infectious Myositides" OR "Myositides, Infectious" OR "Infectious Myositis" OR "Idiopathic Inflammatory Myopathies" OR "Myopathy, Idiopathic Inflammatory" OR "Inflammatory Myopathies, Idiopathic" OR "Myopathies, Idiopathic Inflammatory" OR "Idiopathic Inflammatory Myopathy" OR "Idiopathic Inflammatory Myositis" OR "Inflammatory Myopathy, Idiopathic" OR "IIM")  =32144 items |
|  | #2: TITLE-ABS-KEY ("Dermatomyositis" OR "Polymyositis-Dermatomyositis" OR "Polymyositis Dermatomyositis" OR "Dermatopolymyositis" OR "Dermatomyositis, Adult Type" OR "Adult Type Dermatomyositis" OR "Dermatomyositis, Childhood Type" OR "Childhood Type Dermatomyositis" OR "Juvenile Dermatomyositis" OR "Dermatomyositis, Juvenile" OR "Juvenile Myositis" OR "Myositis, Juvenile")  = 20072 items |
|  | #3: TITLE-ABS-KEY ("Polymyositis" OR "Polymyositides" OR "Myositis, Multiple" OR "Multiple Myositis" OR "Myositides, Multiple" OR "Polymyositis Ossificans" OR "Ossificans, Polymyositis" OR "Polymyositis, Idiopathic" OR "Idiopathic Polymyositides" OR "Idiopathic Polymyositis" OR "Polymyositides, Idiopathic")  =11538 items |
|  | #4: TITLE-ABS-KEY ("Myositis, Inclusion Body" OR "Inclusion Body Myositides" OR "Myositides, Inclusion Body" OR "Inclusion Body Myositis" OR "Inclusion Body Myopathy, Sporadic" OR "Inclusion Body Myositis, Sporadic" OR "Myositis, Inclusion Body, Sporadic" OR "Sporadic Inclusion Body Myositis" OR "Myopathy, Inclusion Body, Sporadic")  =3155 items |
|  | #5: TITLE-ABS-KEY ("Amyopathic dermatomyositis")  =865 items |
|  | #6: TITLE-ABS-KEY ("Antisynthetase syndrome")  =1313 items |
|  | #7: TITLE-ABS-KEY ("RP-ILD" OR "rapidly progressive interstitial lung disease" OR "rapidly progressing interstitial lung disease" OR "rapidly progressive ILD")  =513 items |
|  | #8: #1 OR #2 OR #3 OR #4 OR #5 OR #6  = 49719 items |
|  | #9: #7 AND #8  = **485** items |

Supplementary Table 2 Definitions of Rapidly Progressive Interstitial Lung Disease (RP-ILD) in Included Studies

| No. | Study | Definition |
| --- | --- | --- |
| 1 | Zhang, Y, et al.2022 | RP-ILD was established if the patient showed two or more of the following within 3 months: (1) progressive dyspnea, (2) new ground-glass attenuation (GGA) or consolidation on HRCT, and (3) hypoxemia with > 10 mmHg decrease in arterial oxygen tension(1). The diagnoses of IIM-ILD and RP-ILD were determined by a rheumatologist and a respiratory physician blinded to the result of 18 F-FDG PET/CT. |
| 2 | Li, Y, et al.2021 | Based on the radiological assessment of the chest HRCT result  , RP-ILD was diagnosed later, with the occurrence and development of the disease during 2 to 4 weeks of hospitalization, which was deﬁned as a progressive deterioration of ILD in 3 months combining with rapidly progressive severe dyspnea and hypoxemia, requiring oxygen therapy or ventilator care (2, 3). |
| 3 | Li, Y H, et al.2024 | RP-ILD was defined as ILD showing progression within 1 month of the onset of respiratory symptoms with the presence of any of the following four conditions (4) : (i) acute and progressive worsening of dyspnea requiring hospitalization or supplementary oxygen; (ii) decreases in lung function, including forced vital capacity (FVC) by >10% or reductions in diffusion capacity for carbon monoxide of the lung of over 15% with a decreased FVC; (iii) chest HRCT findings that demonstrate an increase in the extent of interstitial abnormalities to >20%; and (iv) arterial blood gas analysis suggesting respiratory failure or a reduction in oxygen partial pressure reduction of >10 mmHg. |
| 4 | Tsai, H C, et al.2024 | Individuals who developed progressive dyspnea, hypoxemia, and interstitial lesions as demonstrated on the imaging studies within 3 months from the onset of respiratory symptoms were defined as having RPILD (5). |
| 5 | Zhang, Y, et al.2020 | Rapidly progressive ILD (RP-ILD) was defined as worsening of the radiologic interstitial changes with progressive dyspnea and hypoxemia within 3 months after the onset of respiratory symptoms (3). |
| 6 | Liang, J, et al.2021 | A subset of patients with RP-ILD was deﬁned as those presenting with progressive dyspnea and progressive hypoxemia, a worsening of interstitial change on the chest radiograph within 1 month after the initial visit or onset of respiratory symptoms (6-9). |
| 7 | Zhu, D, et al.2021 | The diagnosis of ILD was defined as pulmonary fibrosis detected on chest radiography or high-resolution CT (HRCT) of the lung. All HRCT images were judged by two radiologists. RP-ILD was defined as a previous or con current diagnosis of ILD and acute and progressive worsening of dyspnoea secondary to ILD requiring hospitalization, supplementary oxygen or respiratory failure requiring intubation within 3 months of the diagnosis of ILD (10). |
| 8 | Shi, Y, et al.2024 | Anti-MDA5+DM patients are divided into RP-ILD and non-RP-ILD (including ILD and non-ILD) subgroups according to the presence of any of the following four conditions within one month of the onset of respiratory symptoms: 1) Acute and progressive worsening of dyspnea requiring hospitalization or supplementary oxygen; 2) lung function including forced vital capacity (FVC) decreases by more than 10%, or diffusion capacity for carbon monoxide of the Lung (DLCO) falls over 15% with the decreased FVC; 3) high resolution CT (HRCT) of chest demonstrates that the extent of interstitial abnormalities increased more than 20%; 4) arterial blood gas analysis suggests respiratory failure or the oxy gen partial pressure reduction is greater than 10 mmHg. |
| 9 | Shen, Y W, et al.2020 | Rapidly progressive ILD (RP-ILD) was deﬁned as the deterioration of interstitial lesions (including radiologic interstitial worsening accompanied by progressive dyspnoea and other hypoxemia symptoms) occurring within three months after the occurrence of the ﬁrst respiratory symptoms (10, 11). |
| 10 | Yang, Q, et al.2021 | RPILD was defined as following: deteriorating dyspnea on exertion, decrease in PaO_2_ levels by > 10 mmHg within 4 weeks, or expanding GGO on HRCT within 4 weeks. |
| 11 | Karino, K, et al.2020 | RPILD was defined as a progressive deterioration of ILD with respect to dyspnoea and radiological findings, including chest radio graph and chest high-resolution CT within 3 months from the onset of respiratory symptoms (3, 12-14). |
| 12 | Xu, Y, et al.2016 | The diagnosis of ILD was established by the findings of high-resolution chest computed tomography (HRCT), according to the International Consensus Statement of Idiopathic Pulmonary Fibrosis of the American Thoracic Society (10) and the definition proposed by Suda et al(15). The RP-ILD was defined as a progressive deterioration of ILD within 3 months. |
| 13 | Li, M Q, et al.2023 | The imaging manifestations and lung symptoms had worsened within 3 months or the lung function had markedly worsened since the previous test (e.g., the forced vital capacity decreased by>10% and the partial arterial oxygen pressure decreased by >10 mmHg) (16). |
| 14 | He, W, et al.2024 | RP-ILD was defined as rapid progression of dyspnea symptoms, rapid worsening of HRCT findings, or decrease in partial pressure of oxy gen > 1.33 kPa (10mmHg) within 3 months(17, 18). |
| 15 | Wang, K, et al.2022 | RP-ILD is defined as the presence of one of the following four conditions within one month: 1) Progressive worsening of dyspnea or cough symptoms with a significant decrease in quality of life; 2) A decrease in lung function, including forceful lung volume (FVC) of more than 10% or a decrease in pulmonary diffusion capacity for carbon monoxide (DLCO) of more than 15%; 3) A persistent increase in the degree of interstitial pneumonia on high-resolution CT (HRCT) of the chest; 4) Arterial blood gas analysis demonstrating respiratory failure or a decrease in partial pressure of oxygen more significant than 10 mmHg, independently determined by a senior physician in the rheumatology and respiratory departments. |
| 16 | Zhang, H, et al.2023 | RPILD was defined as progressive dyspnea, hypoxemia, and worsening of the radiological interstitial change at 1 month or deterioration to respiratory failure at 3 months after the onset of respiratory symptoms and no evidence of infections (8, 19). |
| 17 | Zhu, Y, et al.2022 | The diagnosis of RP-ILD was based on both clinical signs and HRCT can features(8, 20).The RP-ILD was defined as the presence of any the following four conditions within one month: 1) dyspnoea or cough symptoms become progressively worse and quality of life is significantly reduced; 2) decreased lung function including forced vital capacity (FVC) decreased by more than 10%, or diffusion capacity for carbon monoxide of the Lung (DLCO) fell over 15% with the de creased FVC; 3) high resolution CT (HRCT) of chest demonstrated that the extent of interstitial pneumonia continues to increase; 4) arterial blood gas analysis suggested respiratory failure or the oxygen partial pressure reduction is greater than 10 mmHg, independently determined by senior physicians in the rheumatology department and the respiratory department. |
| 18 | Li, C, et al.2024 | RPILD was defined as the deterioration of radiologic interstitial lesions >10 % on CT within 1 month, or deterioration to partial pressure of arterial oxygen (PaO_2_) level <60 mmHg within 3 months of the diagnosis of ILD (8, 19). |
| 19 | Liang, J, et al.2021 | A subgroup of RP-ILD patients was considered who presented with progressive dyspnoea and progressive hypoxemia, with acute worsening of interstitial changes on chest radiographs within 1 month after hospitalization or the onset of respiratory symptoms (6, 7, 9). |
| 20 | Sagawa, T, et al.2019 | RP-ILD was defined as follows in this study: presentation of respiratory failure requiring oxygen therapy within 1 month after the DM-associated ILD diagnosis, accompanied by bilateral new ground-glass opacity (GGO) or consolidation, which could not be fully explained by cardiac failure, fluid overload, or infection (21). |
| 21 | Guo, L, et al.2023 | RPILD is defined as either increased level of dyspnea and progression of initial change >10% on CT within 1 month, or deterioration to partial pressure of arterial oxygen (PaO_2_) level <60 mmHg within 3 months of the diagnosis of ILD (8, 19). |

Supplementary Table 3 Results of Newcastle-Ottawa quality assessment Scale for each included study

| No. | Study | Selection | Comparability | Outcome | Total |
| --- | --- | --- | --- | --- | --- |
| 1 | Zhang Y, et al.2022 | **★★★★** | **★★** | **★★★** | 9**★** |
| 2 | Li Y, et al.2021 | **★★★★** | **★★** | **★★** | 8**★** |
| 3 | Li Y H, et al.2024 | **★★★** | **★★** | **★★** | 7**★** |
| 4 | Tsai HC, et al.2024 | **★★★★** | **★★** | **★★★** | 9**★** |
| 5 | Zhang Y, et al.2020 | **★★★★** | **★★** | **★★★** | 9**★** |
| 6 | Liang J, et al.2021 | **★★★★** | **★★** | **★★** | 8**★** |
| 7 | Zhu D, et al.2021 | **★★★★** | **★★** | **★★** | 8**★** |
| 8 | Shi Y, et al.2024 | **★★★** | **★★** | **★★** | 7**★** |
| 9 | Shen YW, et al.2020 | **★★★★** | **★★** | **★★** | 8**★** |
| 10 | Yang Q, et al.2021 | **★★★★** | **★** | **★★** | 7**★** |
| 11 | Karino K, et al.2020 | **★★★★** | **★★** | **★★** | 8**★** |
| 12 | Xu Y, et al.2016 | **★★★** | **★★** | **★★** | 7**★** |
| 13 | Li M, et al.2023 | **★★★★** | **★★** | **★★** | 8**★** |
| 14 | He W, et al.2024 | **★★★★** | **★★** | **★★** | 8**★** |
| 15 | Wang K, et al.2022 | **★★★★** | **★★** | **★★★** | 9**★** |
| 16 | Zhang H, et al.2023 | **★★★★** | **★★** | **★★** | 8**★** |
| 17 | Zhu Y, et al.2022 | **★★★★** | **★★** | **★★** | 8**★** |
| 18 | Li C, et al.2024 | **★★★★** | **★★** | **★★** | 8**★** |
| 19 | Liang J, et al.2021 | **★★★★** | **★★** | **★★** | 8**★** |
| 20 | Sagawa T, et al.2019 | **★★★★** | **★★** | **★★** | 8**★** |
| 21 | Guo L, et al.2023 | **★★★★** | **★★** | **★★** | 8**★** |

Supplementary Figure 1 Plot for the assessment of heterogeneity among the included studies of demographic characteristics (A. Female, B. Disease duration, C. Age) of RP-ILD development in IIM patients through one-by-one elimination method.

Supplementary Figure 2 Plot for the assessment of heterogeneity among the included studies of skin manifestations (A. Gottron papules, B. Heliotrope rash, C. Shawl sign, D. Skin ulceration, E. Periungual erythema) of RP-ILD development in IIM patients through one-by-one elimination method.

Supplementary Figure 3 Plot for the assessment of heterogeneity among the included studies of clinical characteristics (A. Dyspnea, B. Mechanic's hands, C. Fever, D. Dysphagia, E. V sign, F. Muscle weakness, G. Arthritis, H. Pulmonary infection) of RP-ILD development in IIM patients through one-by-one elimination method.

Supplementary Figure 4 Plot for the assessment of heterogeneity among the included studies of IIM subtypes (A. DM, B. CADM) of RP-ILD development in IIM patients through one-by-one elimination method.

Supplementary Figure 5 Plot for the assessment of heterogeneity among the included studies of antibody activity (A. Anti-MDA5, B. Anti-Ro-52, C. Anti-Jo-1, D. Anti-TIF1-γ, E. Anti-ARS, F. Anti-Ku) of RP-ILD development in IIM patients through one-by-one elimination method.

Supplementary Figure 6 Plot for the assessment of heterogeneity among the included studies of serum biomarkers (A. Ferritin(>336ng/ml), B. CK(≥47.5U/L), C. CRP(>0.05mg/L), D. LDH(>280U/L), E. ESR(>21mm/h), F. AST(>36U/L), G. ALT(>27.75U/L)) of RP-ILD development in IIM patients through one-by-one elimination method.

Supplementary Figure 7 Funnel plots of trim-and-fill analysis for odds ratio of RP-ILD development in IIM patients (A: Female, B. Disease duration, C. Age).

Supplementary Figure 8 Funnel plots of trim-and-fill analysis for odds ratio of RP-ILD development in IIM patients (A. Gottron papules, B. Heliotrope rash, C. Shawl sign, D. Skin ulceration, E. Periungual erythema).

Supplementary Figure 9 Funnel plots of trim-and-fill analysis for odds ratio of RP-ILD development in IIM patients (A. Dyspnea, B. Mechanic's hands, C. Fever, D. Dysphagia, E. V sign, F. Muscle weakness, G. Arthritis, H. Pulmonary infection).

Supplementary Figure 10 Funnel plots of trim-and-fill analysis for odds ratio of RP-ILD development in IIM patients (A. DM, B. CADM).

Supplementary Figure 11 Funnel plots of trim-and-fill analysis for odds ratio of RP-ILD development in IIM patients (A. Anti-MDA5, B. Anti-Ro-52, C. Anti-Jo-1, D. Anti-TIF1-γ, E. Anti-ARS, F. Anti-Ku).

Supplementary Figure 12 Funnel plots of trim-and-fill analysis for odds ratio of RP-ILD development in IIM patients (A. Ferritin(>336ng/ml), B. CK(≥47.7U/L), C. CRP(>0.05mg/L), D. LDH(>280U/L), E. ESR(>21mm/h), F. AST(>36U/L), G. ALT(>27.75U/L)).

**References**

1. Akira M, Hamada H, Sakatani M, Kobayashi C, Nishioka M and Yamamoto S. CT findings during phase of accelerated deterioration in patients with idiopathic pulmonary fibrosis. *AJR American journal of roentgenology*. (1997) 168(1):79-83. doi: 10.2214/ajr.168.1.8976924

2. Won Huh J, Soon Kim D, Keun Lee C, Yoo B, Bum Seo J, Kitaichi M, et al. Two distinct clinical types of interstitial lung disease associated with polymyositis-dermatomyositis. *Respiratory medicine*. (2007) 101(8):1761-9. doi: 10.1016/j.rmed.2007.02.017

3. Gono T, Kawaguchi Y, Satoh T, Kuwana M, Katsumata Y, Takagi K, et al. Clinical manifestation and prognostic factor in anti-melanoma differentiation-associated gene 5 antibody-associated interstitial lung disease as a complication of dermatomyositis. *Rheumatology (Oxford, England)*. (2010) 49(9):1713-9. doi: 10.1093/rheumatology/keq149

4. You H, Wang L, Wang J, Lv C, Xu L, Yuan F, et al. Time-dependent changes in RPILD and mortality risk in anti-MDA5+ DM patients: a cohort study of 272 cases in China. *Rheumatology (Oxford, England)*. (2023) 62(3):1216-26. doi: 10.1093/rheumatology/keac450

5. Kobayashi N, Takezaki S, Kobayashi I, Iwata N, Mori M, Nagai K, et al. Clinical and laboratory features of fatal rapidly progressive interstitial lung disease associated with juvenile dermatomyositis. *Rheumatology (Oxford, England)*. (2015) 54(5):784-91. doi: 10.1093/rheumatology/keu385

6. Horiike Y, Suzuki Y, Fujisawa T, Yasui H, Karayama M, Hozumi H, et al. Successful classification of macrophage-mannose receptor CD206 in severity of anti-MDA5 antibody positive dermatomyositis associated ILD. *Rheumatology (Oxford, England)*. (2019) 58(12):2143-52. doi: 10.1093/rheumatology/kez185

7. Hozumi H, Fujisawa T, Nakashima R, Johkoh T, Sumikawa H, Murakami A, et al. Comprehensive assessment of myositis-specific autoantibodies in polymyositis/dermatomyositis-associated interstitial lung disease. *Respiratory medicine*. (2016) 121:91-9. doi: 10.1016/j.rmed.2016.10.019

8. Sato S, Hirakata M, Kuwana M, Suwa A, Inada S, Mimori T, et al. Autoantibodies to a 140-kd polypeptide, CADM-140, in Japanese patients with clinically amyopathic dermatomyositis. *Arthritis and rheumatism*. (2005) 52(5):1571-6. doi: 10.1002/art.21023

9. Abe Y, Matsushita M, Tada K, Yamaji K, Takasaki Y and Tamura N. Clinical characteristics and change in the antibody titres of patients with anti-MDA5 antibody-positive inflammatory myositis. *Rheumatology (Oxford, England)*. (2017) 56(9):1492-7. doi: 10.1093/rheumatology/kex188

10. American Thoracic Society. Idiopathic pulmonary fibrosis: diagnosis and treatment. International consensus statement. American Thoracic Society (ATS), and the European Respiratory Society (ERS). *American journal of respiratory and critical care medicine*. (2000) 161(2 Pt 1):646-64. doi: 10.1164/ajrccm.161.2.ats3-00

11. Lucà S, Pagliuca F, Perrotta F, Ronchi A, Mariniello DF, Natale G, et al. Multidisciplinary Approach to the Diagnosis of Idiopathic Interstitial Pneumonias: Focus on the Pathologist's Key Role. *International journal of molecular sciences*. (2024) 25(7) doi: 10.3390/ijms25073618

12. Xu Y, Yang CS, Li YJ, Liu XD, Wang JN, Zhao Q, et al. Predictive factors of rapidly progressive-interstitial lung disease in patients with clinically amyopathic dermatomyositis. *Clinical rheumatology*. (2016) 35(1):113-6. doi: 10.1007/s10067-015-3139-z

13. Gono T, Sato S, Kawaguchi Y, Kuwana M, Hanaoka M, Katsumata Y, et al. Anti-MDA5 antibody, ferritin and IL-18 are useful for the evaluation of response to treatment in interstitial lung disease with anti-MDA5 antibody-positive dermatomyositis. *Rheumatology (Oxford, England)*. (2012) 51(9):1563-70. doi: 10.1093/rheumatology/kes102

14. Ishigaki K, Maruyama J, Hagino N, Murota A, Takizawa Y, Nakashima R, et al. Skin ulcer is a predictive and prognostic factor of acute or subacute interstitial lung disease in dermatomyositis. *Rheumatology international*. (2013) 33(9):2381-9. doi: 10.1007/s00296-013-2735-y

15. Suda T, Fujisawa T, Enomoto N, Nakamura Y, Inui N, Naito T, et al. Interstitial lung diseases associated with amyopathic dermatomyositis. *The European respiratory journal*. (2006) 28(5):1005-12. doi: 10.1183/09031936.06.00038806

16. Travis WD, Costabel U, Hansell DM, King TE, Jr., Lynch DA, Nicholson AG, et al. An official American Thoracic Society/European Respiratory Society statement: Update of the international multidisciplinary classification of the idiopathic interstitial pneumonias. *American journal of respiratory and critical care medicine*. (2013) 188(6):733-48. doi: 10.1164/rccm.201308-1483ST

17. So J, So H, Wong VT, Ho R, Wu TY, Wong PC, et al. Predictors of rapidly progressive interstitial lung disease and mortality in patients with autoantibodies against melanoma differentiation-associated protein 5 dermatomyositis. *Rheumatology (Oxford, England)*. (2022) 61(11):4437-44. doi: 10.1093/rheumatology/keac094

18. Kishaba T. Acute Exacerbation of Idiopathic Pulmonary Fibrosis. *Medicina (Kaunas, Lithuania)*. (2019) 55(3) doi: 10.3390/medicina55030070

19. Moghadam-Kia S, Oddis CV, Sato S, Kuwana M and Aggarwal R. Anti-Melanoma Differentiation-Associated Gene 5 Is Associated With Rapidly Progressive Lung Disease and Poor Survival in US Patients With Amyopathic and Myopathic Dermatomyositis. *Arthritis care & research*. (2016) 68(5):689-94. doi: 10.1002/acr.22728

20. Abe Y, Kusaoi M, Tada K, Yamaji K and Tamura N. Successful treatment of anti-MDA5 antibody-positive refractory interstitial lung disease with plasma exchange therapy. *Rheumatology (Oxford, England)*. (2020) 59(4):767-71. doi: 10.1093/rheumatology/kez357

21. Collard HR, Ryerson CJ, Corte TJ, Jenkins G, Kondoh Y, Lederer DJ, et al. Acute Exacerbation of Idiopathic Pulmonary Fibrosis. An International Working Group Report. *American journal of respiratory and critical care medicine*. (2016) 194(3):265-75. doi: 10.1164/rccm.201604-0801CI
